# Endovascular thrombectomy activity as a candidate marker of organised acute stroke-service capacity: a global burden of disease-based European panel study

**DOI:** 10.1093/esj/aakag121

**Published:** 2026-10-15

**Authors:** Felix Gunzer, Fatih Dudic, Taylan D Kuzu, Benedikt Frank, Natalie van Landeghem, Michael Forsting, Yan Li, Johannes Haubold

**Affiliations:** Institute of Diagnostic and Interventional Radiology and Neuroradiology, University Hospital Essen, Essen, Germany; Institute of Diagnostic and Interventional Radiology and Neuroradiology, University Hospital Essen, Essen, Germany; University of Cologne, Faculty of Medicine and University Hospital Cologne, Department of Neurology, Cologne, Germany; Department of Neurology and Center for Translational Neuro- and Behavioral Sciences (C-TNBS), University Hospital Essen, University Duisburg-Essen, Essen, Germany; Institute of Diagnostic and Interventional Radiology and Neuroradiology, University Hospital Essen, Essen, Germany; Institute of Diagnostic and Interventional Radiology and Neuroradiology, University Hospital Essen, Essen, Germany; Institute of Diagnostic and Interventional Radiology and Neuroradiology, University Hospital Essen, Essen, Germany; Institute of Diagnostic and Interventional Radiology and Neuroradiology, University Hospital Essen, Essen, Germany

**Keywords:** cerebral infarction, endovascular procedures, global burden of disease, health services accessibility, ischemic stroke, thrombectomy

## Abstract

**Introduction:**

Acute stroke-care organisation might contribute to variation in ischaemic stroke outcomes across Europe. We assessed whether national endovascular thrombectomy activity was associated with Global Burden of Disease–derived ischaemic stroke burden across European and adjacent countries.

**Patients and methods:**

We conducted a repeated cross-sectional ecological analysis of a balanced three-wave panel of 39 countries (2016, 2019 and 2020) using a consistent thrombectomy data source. The primary outcome was the ischaemic stroke mortality-to-incidence ratio. We used a within–between model with wave fixed effects, adjusted for Socio-Demographic Index and population aged 65 years or older.

**Results:**

Between countries, higher thrombectomy activity was associated with a lower mortality-to-incidence ratio (fully standardised β = −0.42, 95% confidence interval −0.79 to −0.05; *P* = .027), whereas no within-country association was detected (β = +0.10, −0.07 to +0.26). The between-country estimate attenuated after joint adjustment for health expenditure and Universal Health Coverage (β = −0.28; *P* = .051) and after adjustment for stroke incidence (β = −0.33; *P* = .061). In leave-one-country-out analyses, *P* values ranged from .007 to .061.

**Discussion:**

The between-country estimate is more vulnerable to unmeasured national differences and could not be separated from health-system resources. This ecological study cannot estimate the causal effect of increasing thrombectomy provision.

**Conclusion:**

National thrombectomy activity distinguished countries with lower ischaemic stroke mortality relative to incidence but did not track within-country change. It remains a candidate marker for cross-country comparison, not an established indicator for monitoring change, and should be interpreted alongside directly measured quality-of-care indicators.

## Graphical abstract

**Graphical Abstract:**
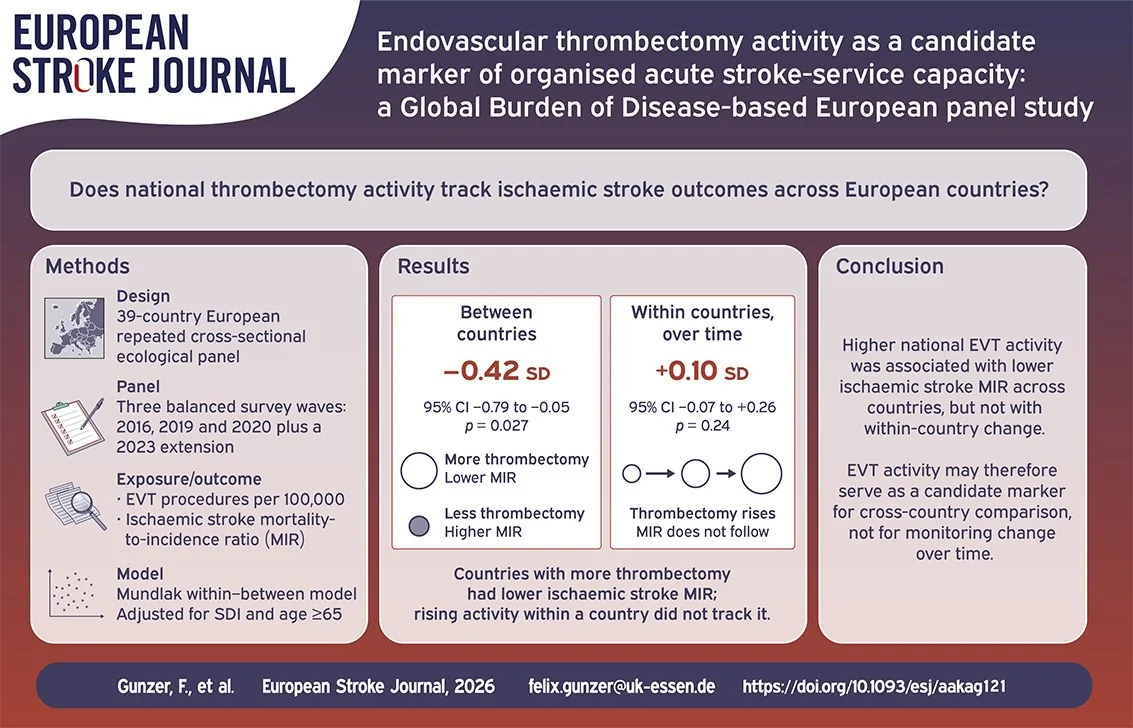

## Introduction

Stroke is the second leading cause of death and the third leading cause of disability worldwide, accounting for approximately 6.6 million deaths and 143 million disability-adjusted life-years (DALYs) annually.^1^ The burden falls disproportionately on low- and middle-income countries, but substantial variation in mortality-related ischaemic stroke burden also persists among European and adjacent countries, where many individual components of acute stroke care are widely available.^2^^,^^3^

Endovascular thrombectomy (EVT) sits at the apex of the acute stroke-care chain: it markedly reduces disability after large-vessel-occlusion ischaemic stroke^4–7^ and European guidelines recommend it within 6 hours of onset and in selected patients up to 24 hours,^8^ but at population level it presupposes an upstream pathway of prehospital triage, vascular imaging, transfer and organised stroke-unit care.^9^^,^^10^ European standards define organised stroke-unit care and set a coverage target for it,^11^ but cross-national survey measures of stroke-unit availability and access differ in ascertainment and granularity, whereas the thrombectomy procedure is counted the same way in every centre that performs it. National EVT activity may therefore be the more comparable measure across countries, and that narrow comparability is why we examine it as a candidate marker of organised acute stroke-service capacity. Because these workflows also serve the nontarget majority of ischaemic strokes, EVT activity plausibly reflects whole-system quality, not just the eligible minority.

We therefore examined two related questions. First, whether the association between national EVT activity and stroke burden was more evident for mortality-related outcomes than for disability-only burden such as years lived with disability (YLDs). Second, whether EVT estimates differed in pattern from those of other acute-care indicators (intravenous thrombolysis [IVT] rate, computed tomography [CT] scanner density, stroke unit density) and broader macro-system indicators (national health expenditure, Universal Health Coverage [UHC] service coverage, the Gini index)^12^^,^^13^ assessed within the same modelling framework.

We addressed these questions in a repeated cross-sectional panel of 39 European and adjacent countries observed in 2016, 2019 and 2020, extended to 2023 for the 27 countries reporting then, with Global Burden of Disease (GBD) estimates of ischaemic stroke burden as outcomes; the primary endpoint was the ischaemic stroke mortality-to-incidence ratio (MIR) and the primary model a within–between decomposition adjusted for socioeconomic development and age structure.

## Patients and methods

### Study design

We carried out a repeated cross-sectional ecological analysis at the country level across 39 European and adjacent countries, examining cross-national associations between national EVT activity and GBD 2023 release estimates of ischaemic stroke burden. The primary panel was the balanced three-wave Aguiar specification (2016, 2019, 2020; 117 country-year observations, single EVT data source), so the country-mean EVT exposure was calculated over the same three waves for every country. The four-wave extension adds the 2023 European Stroke Organisation (ESO) Stroke Service Tracker wave (144 country-year observations; 27 of 39 countries reporting) and is secondary because of its different data source and incomplete coverage. The primary endpoint was ischaemic stroke MIR, a population-level summary built from modelled GBD rates rather than an observed case-fatality rate; secondary endpoints were ischaemic stroke years of life lost (YLLs), DALYs, and YLDs per 100,000 population, with the all-stroke endpoints in the Supplementary Material. The primary model was a within–between decomposition (Mundlak parameterisation) with wave fixed effects (FE) and country-clustered standard errors (SE). Reporting follows the protocols of Strengthening the Reporting of Observational Studies in Epidemiology (STROBE)^14^ and Guidelines for Accurate and Transparent Health Estimates Reporting (GATHER).^15^

### Data sources

National EVT rates (procedures per 100,000 population/year) for waves 1–3 (2016, 2019, 2020) were taken from the Aguiar de Sousa et al. surveys.^9^^,^^16^ Wave 4 (2023) rates were derived from the ESO Stroke Action Plan for Europe Stroke Service Tracker,^17^ which reports mechanical thrombectomy (MT) as a percentage of ischaemic stroke admissions. The 2023 country-level aggregates were obtained from the European Stroke Organisation under written approval from the Stroke Action Plan committee, which is also the condition on their reuse. We converted the percentages to per-100,000 using country-matched admissions and population values (conversion formula and edge cases in the Supplementary Methods). Wave 4 yielded valid EVT values for 27 of 39 panel countries; all 12 exclusions rest on a missing MT percentage or admission count, none on a reported zero. The resulting exposure is population-based activity, not the Stroke Action Plan for Europe key performance indicator, which is MT as a percentage of ischaemic strokes;^11^ only the population rate exists across all four waves. United Kingdom constituent nations were aggregated to a single GBR value using admission-weighted percentages. Admission counts and MT percentages are reported by each country’s coordinator; where registry coverage is incomplete the conversion assumes the percentage represents the reported admissions, so those 2023 rates are country-level estimates rather than fully ascertained national rates (Table S17).

Country-level stroke burden was obtained from the GBD 2023 release (deaths, incidence, YLLs, YLDs, DALYs per 100,000; Rate metric, both sexes, all ages; ischaemic and all-stroke separately).^1^ The MIR was defined as the ratio of the death rate to the incidence rate. All four waves use this single release, so within-country comparisons are free of release-to-release revisions.

The CT scanner density was compiled from the IAEA IMAGINE (IAEA Medical imAGIng and Nuclear mEdicine global resources database) database,^18^ OECD Health Statistics^19^ and the World Health Organization (WHO) Global Health Observatory,^13^ in that priority order, with linear interpolation across missing intermediate years. The comparator and adjustment covariates—IVT rate, stroke unit density, total health expenditure (THE) per capita, UHC service coverage and the Gini index—come from ESO surveys, the WHO Global Health Expenditure Database, WHO/World Bank UHC tracking and the World Bank Development Indicators.^12^ Intravenous thrombolysis (IVT) 2023 was derived from the Tracker like EVT 2023, with the same source change; stroke unit density is a country-level snapshot.

The Socio-Demographic Index (SDI, GBD 2023 release) and the proportion of the population aged 65 years or older (World Bank Development Indicators) were the adjustment variables in all primary models.

### Statistical analysis

#### Primary model

For each country *i* at wave *t*, the EVT rate was decomposed into a country-level time-averaged mean $ {\overline{x}}_i $ and a deviation $ {x}_{it}-{\overline{x}}_i $:


$$ {y}_{it}=\alpha +{\beta}_W\left({x}_{it}-{\overline{x}}_i\right)+{\beta}_B{\overline{x}}_i+{\gamma}^{\prime }{\boldsymbol{z}}_{it}+{\delta}_t+{\varepsilon}_{it} $$

where ${y}_{it}$ is the endpoint, ${\beta}_W$ and ${\beta}_B$ are the within- and between-country EVT associations, ${\boldsymbol{z}}_{it}$ contains the SDI and age65+ adjustment covariates, and ${\delta}_t$ is the wave fixed effect, absorbing time trends common to all countries. The model was fitted by pooled ordinary least squares (OLS), with the covariates entering undecomposed and without a country random intercept; SE were clustered at the country level (39 clusters) with finite-sample correction. Two alternative forms, with SDI and age65+ decomposed like EVT and with a country random intercept added, are reported in Table S16. The between- and within-country EVT coefficients for the four ischaemic endpoints are presented in fully standardised form (per 1 standard deviation [SD] higher EVT, expressed in SD of the endpoint), both SDs being the pooled SD over all country-year observations, applied to the between- and within-country terms alike. Because within-country variation is small relative to between-country variation in this panel, we also report the smallest within-country effect the primary model could have detected with 80% power.

#### Comparator and adjusted-EVT models

In the same framework, six additional exposures were each standardised per 1 SD and tested against ischaemic MIR: IVT rate, CT scanner density, stroke unit density, THE per capita, UHC service coverage and the Gini index. Intravenous thrombolysis (IVT) enters as a single country-level mean of the 2019 and 2020 survey values, so that and stroke unit density are between-only comparators; IVT 2023 was confined to extension analyses. To assess whether the EVT–MIR association reflected broader resource gradients, we fitted four adjusted specifications: SDI replaced by THE; by UHC; THE and UHC jointly (interpreted cautiously, as THE, UHC, and SDI are collinear); and IVT, stroke unit density and CT added alongside SDI as an acute-process adjustment.

#### Sensitivity and extension analyses

Selection on 2023 reporting was addressed by restricting the extension to the 27 reporters and by comparing reporters with nonreporters. Because MIR is a ratio of two modelled GBD rates, and because stroke incidence could in principle raise both EVT per head of population and the ratio’s denominator, we also fitted the primary model with the death rate and the incidence rate as separate outcomes, with MIR adjusted for the incidence rate, and with the log death rate adjusted for the log incidence rate (Table S15). Because GBD’s mortality and incidence models draw on sociodemographic covariates, the confounder-set variants include specifications without SDI. Supporting specifications—pooled OLS, two-way FE, first differences, FE excluding 2020, confounder-set variants S1–S7 (+Gini; +IVT + stroke units; UHC-only; THE-only; population-weighted; age65+ only; unadjusted) and influence diagnostics (Cook’s distance and leave-one-country-out)—are reported in the Supplementary Methods and Supplementary Tables. A single primary hypothesis was prespecified, the between-country EVT association with ischaemic MIR; every other estimate is secondary, unadjusted for multiplicity and exploratory. The prespecified analysis hierarchy was amended before inspection of outcome regression results, as described next.

#### Changes from the protocol

The protocol prespecified the unbalanced four-wave analysis as primary and the balanced three-wave panel as a sensitivity; after data assembly but before any outcome regression was inspected, we exchanged the two, because the source change and incomplete 2023 reporting compromised the between-country EVT mean as a uniform construct. A second amendment and the rationale are in the Supplementary Methods; both are logged in the repository history.

#### Software

All analyses were done in Python 3.13 (statsmodels, linearmodels, pandas, numpy, matplotlib, geopandas); the code, with pinned requirements, is publicly available in Mendeley Data (DOI: 10.17632/r9p47jkz8w.1).

### Ethics

The analysis used aggregate country-year data only. Use of the nonpublic ESO Stroke Service Tracker data was authorised by the ESO Stroke Action Plan committee; all other sources are public. Ethics approval and informed consent were not required, and no human participants were enrolled (prespecified in the protocol).

## Results

The primary balanced panel comprised 39 European and adjacent countries observed in 2016, 2019 and 2020 (117 country-year observations, single EVT data source); the four-wave extension added the 2023 ESO Stroke Service Tracker wave for 27 of them (144 observations). Figure 1 maps country-mean EVT activity and ischaemic stroke MIR on the three-wave base. Median EVT rate was 3.7 per 100,000 (interquartile range [IQR] 0.5–7.1) in 2016 and 13.5 per 100,000 (IQR 8.9–16.7) in 2023, and median ischaemic stroke MIR was 0.52 in 2016 against 0.43 in 2023 (Table 1). These wave medians are not a longitudinal trend: the 27 countries reporting in 2023 already had higher EVT activity and lower MIR than the 12 nonreporters beforehand (Table S7), and within those 27 median EVT rose from 4.8 to 13.5 per 100,000 while median MIR moved only from 0.44 to 0.43.

**Figure 1:**
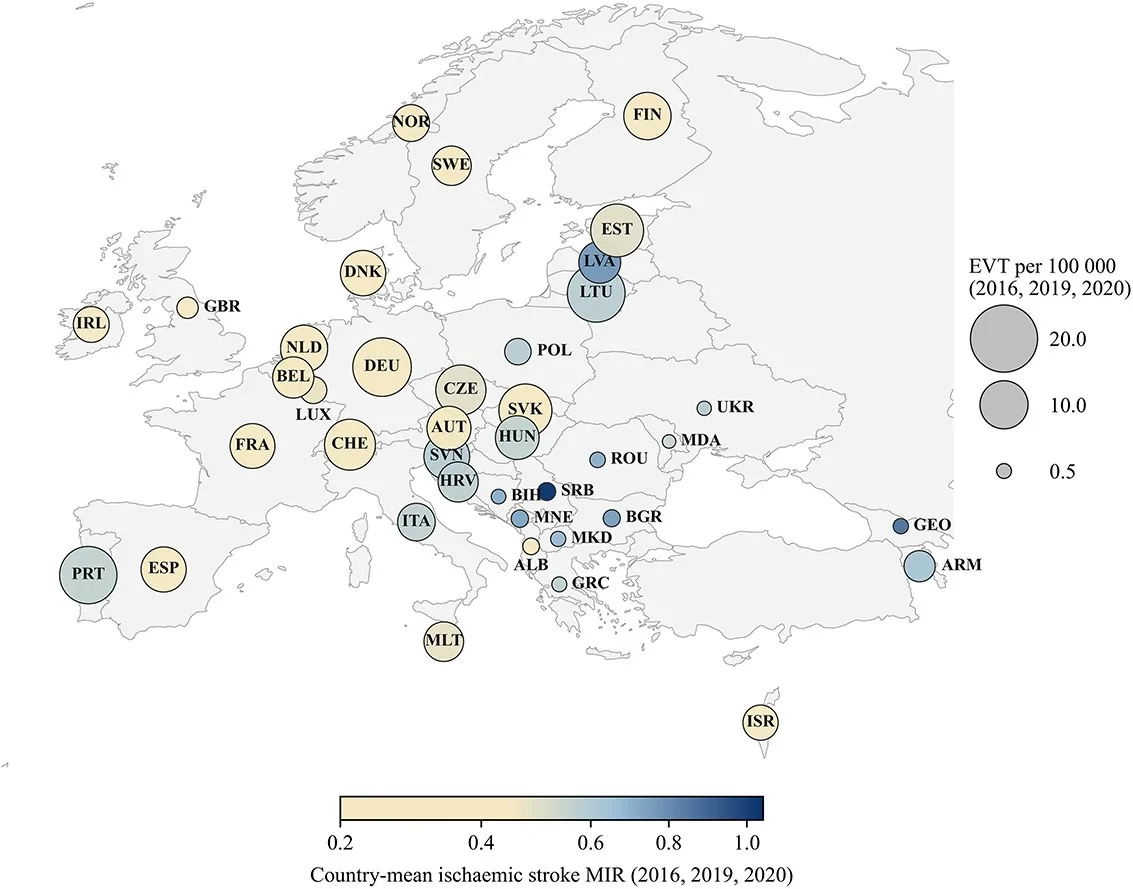
Country-level summary of EVT activity and ischaemic stroke MIR across the 39 panel countries, averaged over the three-wave primary panel (2016, 2019 and 2020). Circle area is proportional to country-mean EVT rate per 100,000 population (legend right); fill colour shows country-mean ischaemic stroke MIR on a continuous yellow-to-blue scale (yellow = lower, blue = higher MIR; legend below). Country labels are ISO 3166-1 alpha-3 codes; light-grey country outlines provide geographic reference. Descriptive figure: no statistical test was applied. Thirty-nine countries, 117 country-year observations. Drawn in Python with matplotlib and geopandas from the analysis outputs; country boundaries from natural earth 1:50 m. No image was acquired or edited outside these tools. Abbreviations: EVT = endovascular thrombectomy; MIR = mortality-to-incidence ratio.

**Table 1:** Panel descriptive statistics.

**Variable**	**Overall**	**2016**	**2019**	**2020**	**2023**
**EVT rate per 100,000**	7.58 (1.28–12.65)	3.72 (0.46–7.12)	9.05 (1.79–12.86)	9.23 (1.18–12.77)	13.46 (8.87–16.66)
**CT scanners per 100,000**	1.87 (1.35–2.71)	1.72 (1.11–2.34)	1.81 (1.17–2.62)	1.91 (1.17–2.76)	2.42 (1.83–3.09)
**SDI (0–1)**	0.81 (0.76–0.85)	0.79 (0.75–0.84)	0.80 (0.76–0.85)	0.81 (0.77–0.85)	0.83 (0.77–0.86)
**Population aged ≥65 years (%)**	19.18 (16.75–20.44)	18.44 (15.18–19.34)	19.06 (16.49–20.18)	19.37 (16.93–20.48)	20.16 (18.90–20.87)
**All-stroke MIR**	0.53 (0.44–0.66)	0.58 (0.46–0.71)	0.59 (0.45–0.68)	0.54 (0.43–0.69)	0.49 (0.43–0.55)
**All-stroke YLLs per 100,000**	1329.85 (895.72–2783.69)	1479.04 (950.28–2928.72)	1344.73 (885.88–2741.24)	1391.39 (861.13–2742.36)	1254.87 (895.31–2235.25)
**All-stroke YLDs per 100,000**	271.59 (211.22–314.33)	263.52 (202.07–308.87)	270.28 (209.72–315.52)	272.40 (211.52–318.37)	273.27 (216.23–313.62)
**All-stroke DALYs per 100,000**	1602.16 (1126.76–3044.40)	1717.87 (1170.50–3206.45)	1636.14 (1132.52–3029.22)	1683.14 (1103.42–3034.94)	1511.42 (1129.55–2563.23)
**Ischaemic stroke MIR**	0.48 (0.38–0.59)	0.52 (0.40–0.60)	0.49 (0.38–0.59)	0.47 (0.37–0.60)	0.43 (0.38–0.52)
**Ischaemic stroke YLLs per 100,000**	885.22 (483.61–1691.55)	875.89 (526.05–1910.09)	903.39 (490.56–1607.42)	949.06 (481.33–1758.24)	858.04 (483.07–1398.94)
**Ischaemic stroke YLDs per 100,000**	215.72 (165.67–255.99)	210.42 (159.60–240.95)	217.13 (166.81–254.62)	219.25 (168.68–257.77)	219.77 (170.77–259.38)
**Ischaemic stroke DALYs per 100,000**	1081.34 (682.40–1920.22)	1038.18 (697.47–2145.63)	1121.53 (678.03–1844.62)	1168.32 (682.43–2005.55)	1071.22 (691.36–1638.13)
**n countries**	39	39	39	39	27
**n observations**	144	39	39	39	27

Country-year median values (IQR) across the four waves of the panel. The 2016, 2019 and 2020 columns cover all 39 panel countries; the 2023 column covers the 27 countries reporting to the ESO Stroke Service Tracker. The CT scanner density was linearly interpolated where annual source data were unavailable, and per-variable counts may fall short of the panel *n* where a country lacks a given variable in a given wave. Abbreviations: CT = computed tomography; DALYs = disability-adjusted life-years; ESO = European Stroke Organisation; EVT = endovascular thrombectomy; IQR = interquartile range; IVT = intravenous thrombolysis; MIR = mortality-to-incidence ratio; SDI = Socio-Demographic Index; YLDs = years lived with disability; YLLs = years of life lost.

### Endovascular thrombectomy activity and GBD-derived ischaemic stroke burden

In the primary three-wave within–between model with wave fixed effects, higher between-country EVT activity was associated with lower ischaemic stroke MIR (fully standardised β_between = −0.42 SD MIR per SD EVT, 95% confidence interval [CI] −0.79 to −0.05; cluster-robust *P* = .027; 39 countries, 117 observations; Figure 2, Table 2). The within-country EVT term was not statistically distinguishable from zero once common time trends were absorbed (β_within = +0.10, 95% CI −0.07 to +0.26, *P* = .24). The 80% power threshold was 0.23 SD, more than half the between-country estimate, indicating limited sensitivity to smaller within-country associations. The between-country direction was consistent across mortality-related endpoints (YLLs β_between = −0.40, *P* = .059; DALYs −0.38, *P* = .075) but was null for YLDs (−0.02, *P* = .92). Within-country estimates were near zero for all four endpoints.

**Figure 2:**
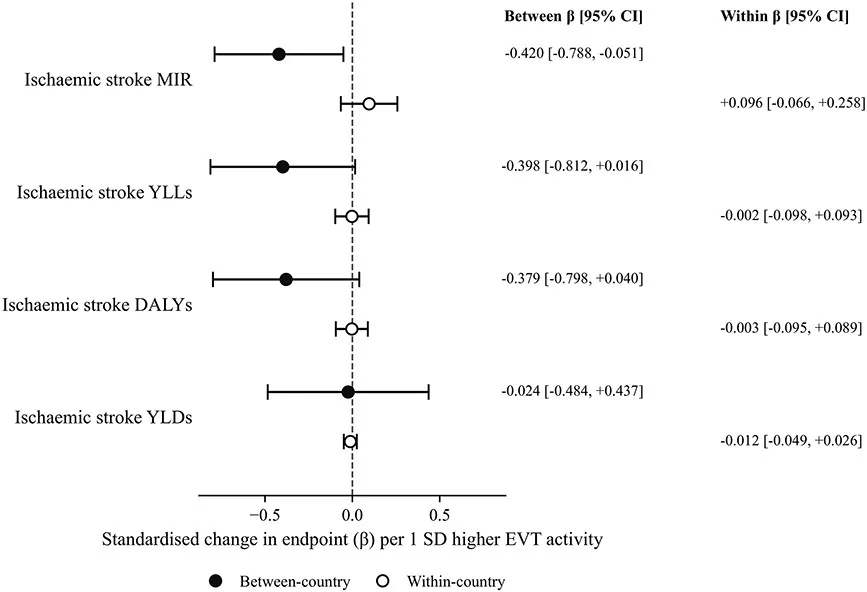
Between- and within-country associations of EVT activity with GBD-derived ischaemic stroke burden, fully standardised (difference in outcome SDs per 1 SD higher EVT activity). Filled circles: between-country component; open circles: within-country component. Within–between decomposition with wave fixed effects, adjusted for SDI and proportion aged ≥65 years; country-clustered robust SE (finite sample–corrected). Balanced three-wave Aguiar primary panel (39 countries, 117 country-year observations). Drawn in Python with matplotlib from the analysis outputs; no image was acquired or edited outside these tools. Abbreviations: DALYs = disability-adjusted life-years; EVT = endovascular thrombectomy; GBD = Global Burden of Disease; MIR = mortality-to-incidence ratio; SD = standard deviation; SDI = Socio-Demographic Index; SE = standard error; YLDs = years lived with disability; YLLs = years of life lost.

**Table 2:** Within–between estimation with wave fixed effects: EVT rate and ischaemic stroke burden endpoints.

**Endpoint**	**Between β (95% CI)**	**Between *P***	**Within β (95% CI)**	**Within *P***	***n* Obs**	***n* Countries**
**Ischaemic stroke MIR (primary)**	–0.0127 (–0.0239, –0.0016)	.027	0.0029 (–0.0020, 0.0078)	.24	117	39
**Ischaemic stroke YLLs per 100,000**	–66.5489 (–135.7267, 2.6290)	.059	–0.3918 (–16.3477, 15.5642)	.96	117	39
**Ischaemic stroke DALYs per 100,000**	–66.8303 (–14.6315, 6.9710)	.075	–0.5294 (–16.6697, 15.6110)	.95	117	39
**Ischaemic stroke YLDs per 100,000**	–0.2814 (–5.7007, 5.1379)	.92	–0.1376 (–0.5762, 0.3011)	.53	117	39
**Four-wave extension sensitivity (MIR only)**						
**Ischaemic stroke MIR (4-wave extension, unbalanced)**	–0.0098 (–0.0190, –0.0007)	.036	0.0025 (–0.0024, 0.0075)	.31	144	39
**Ischaemic stroke MIR (balanced 27, all 4 waves)**	–0.0056 (–0.0138, 0.0027)	.18	0.0009 (–0.0032, 0.0049)	.66	108	27

Each row is a within–between linear panel model with wave fixed effects, adjusted for SDI and the proportion aged ≥65 years; standard errors are country-clustered and finite-sample-corrected. Coefficients are in raw outcome units per +1 procedure per 100,000 of EVT (Table 3 expresses the primary ischaemic-MIR estimate on the fully standardised scale, with 1 EVT SD = 5.97 per 100,000). The upper block covers the balanced three-wave Aguiar primary panel (39 countries, 117 country–year observations; four ischaemic endpoints). The lower block reports 2 four-wave extension sensitivity analyses for ischaemic MIR: the unbalanced four-wave fit (39 countries, 144 observations) and a balanced subrestriction to the 27 wave-4 reporters (108 observations); restricting to wave-4 reporters reduces precision and removes the reporter-versus-nonreporter contrast; in this subset the point estimate was attenuated (descriptive reporter comparison in Table S7). All-stroke endpoints are reported in the Supplementary Material. Abbreviations: CI = confidence interval; DALYs = disability-adjusted life-years; EVT = endovascular thrombectomy; MIR = mortality-to-incidence ratio; SD = standard deviation; YLDs = years lived with disability; YLLs = years of life lost.

In the four-wave extension (144 observations), the between-country estimate was similar (β_between = −0.38, 95% CI −0.73 to −0.03, *P* = .036) and the within-country term still null (*P* = .31). Because the data source changes only in 2023, source and period effects are not separable in the extension, and restricting it to the 27 reporters returned a nonsignificant coefficient (−0.006, *P* = .18; Table 2, lower panel, in raw outcome units). The reporter-versus-nonreporter difference (Table S7) is itself a plausible selection bias, so the extension corroborates the three-wave primary only weakly.

### Comparator exposures: acute-care and macro-system indicators

In the same three-wave framework, among acute stroke-care indicators only EVT had a between-country 95% CI excluding the null for ischaemic stroke MIR; IVT rate (β_between = +0.08, 95% CI −0.30 to +0.46, *P* = .67), CT scanner density (+0.10, −0.29 to +0.48, *P* = .60) and stroke unit density (+0.18, −0.05 to +0.42, *P* = .13) all showed null patterns (Figure 3A). No between-exposure test was performed, and the comparators rest on heterogeneous samples (EVT: 117/39; IVT: 72/36, 2019/2020 only; CT: 117/39, partly interpolated; stroke unit density: 74/37, time-invariant).

**Figure 3:**
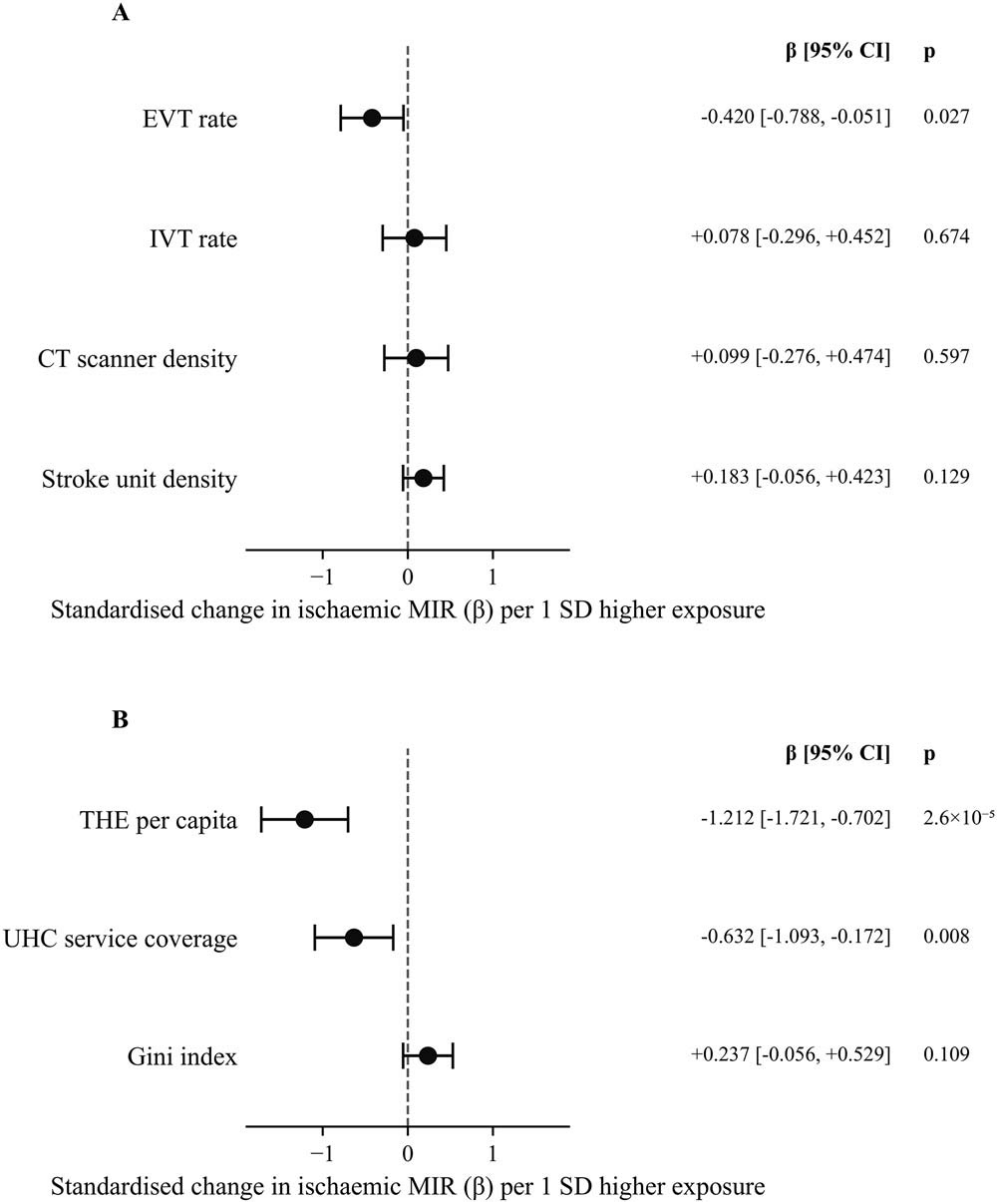
Between-country associations of stroke-system and macro-system indicators with ischaemic stroke MIR. (A) Acute stroke-care process and capacity indicators. (B) Macro-system indicators. Each exposure is z-standardised within its own analytic sample and THE outcome is also z-standardised, so estimates are SD of MIR per 1 SD higher exposure. Between-country fits with wave fixed effects and finite-sample-corrected country-clustered SE on THE balanced three-wave Aguiar primary panel. Analytic samples (observations/ countries): EVT 117/39, IVT 72/36 (2019/2020 only), CT 117/39 (partly interpolated), stroke unit density 74/37 (snapshot); THE 111/37; UHC 111/37; Gini 110/37. The IVT rate and stroke unit density entered as time-invariant country-level comparators and therefore had no estimable within-country component. Coefficients are reported per exposure and are not formally compared. Drawn in Python with matplotlib from THE analysis outputs; no image was acquired or edited outside these tools. Abbreviations: CI = confidence interval; CT = computed tomography; EVT = endovascular thrombectomy; IVT = intravenous thrombolysis; MIR = mortality-to-incidence ratio; SD = standard deviation; SE = standard error; THE = total health expenditure per capita; UHC = Universal Health Coverage service coverage index.

Among broader macro-system indicators (Figure 3B), total health expenditure per capita (β_between = −1.21, 95% CI −1.72 to −0.70, *P* = 2.6 × 10^−5^) and UHC service coverage (−0.63, −1.09 to −0.17, *P* = .009) had larger fully standardised between-country point estimates in their analytic samples, and the Gini index a positive one whose 95% CI included the null (+0.24, −0.06 to +0.53, *P* = .11). These reproduce the well-described between-country gradient of national health-system resources with stroke mortality.

### Adjustment for health-system resources and acute-care indicators

Adjustment for macro-system resources attenuated the EVT–MIR association in the three-wave primary (Table 3). Each adjusted specification is paired with a same-sample baseline (the primary model on the reduced sample), so differences reflect adjustment rather than sample restriction. On the macro-system sample (111 observations, 37 countries), the SDI + age 65+ baseline gives β_between = −0.45 (*P* = .030); replacing SDI with THE attenuated this to −0.29 (*P* = .029), replacing SDI with UHC to −0.32 (*P* = .054), and joint adjustment for THE and UHC to −0.28 (*P* = .051), with the 95% CI including the null: an attenuation of about one-third relative to that baseline, which is the pattern expected if national health-system resources confound part of the between-country association. On the acute-process sample (72 observations, 36 countries), the baseline gives β_between = −0.36 (*P* = .051) and adding IVT, stroke unit density and CT moved it to −0.56 (*P* = .014); these indicators did not attenuate the between-country EVT coefficient in this sample.

**Table 3:** Adjusted EVT–ischaemic MIR estimates.

**Model**	**Adjustment set**	**Between β (fully standardised, 95% CI)**	**Between *P***	**Within β (fully standardised, 95% CI)**	**Within *P***	***n* Obs**	***n* Countries**
**Primary model**	SDI + age 65+ (baseline, full primary sample)	−0.42 (−0.79, −0.05)	.027	0.10 (−0.07, 0.26)	.24	117	39
**Same-sample baseline (acute-process sample)**	SDI + age 65+ on the IVT/SU/CT-complete sample	−0.36 (−0.73, 0.00)	.051	0.10 (−0.00, 0.21)	.057	72	36
**Acute-process adjusted**	IVT rate + stroke unit density + CT + SDI + age 65+	−0.56 (−0.99, −0.12)	.014	0.11 (−0.01, 0.23)	.061	72	36
**Same-sample baseline (macro-system sample)**	SDI + age 65+ on the THE/UHC-complete sample	−0.45 (−0.86, −0.05)	.03	0.13 (−0.02, 0.28)	.086	111	37
**THE-adjusted**	THE per capita + age 65+ (no SDI)	−0.29 (−0.55, −0.03)	.029	0.22 (0.04, 0.40)	.016	111	37
**UHC-adjusted**	UHC service coverage + age 65+ (no SDI)	−0.32 (−0.64, 0.01)	.054	0.13 (−0.03, 0.30)	.12	111	37
**Macro-system adjusted**	THE per capita + UHC service coverage + age 65+ (no SDI)	−0.28 (−0.56, 0.00)	.051	0.22 (0.03, 0.41)	.026	111	37

Standardised change in ischaemic stroke MIR per +1 SD higher EVT activity, in the primary specification and under acute-process and macro-system adjustment. Coefficients are fully standardised (SD of outcome per SD of exposure) and match the scale used in the Abstract, Results and Figure 3 (primary between-country β = −0.42). Each adjusted specification is paired with a same-sample baseline that refits the primary SDI + age 65+ model on the reduced analytic sample, so any change relative to that baseline reflects covariate adjustment rather than sample restriction. The joint THE + UHC specification should be interpreted with caution because of collinearity between THE, UHC and SDI. Standard errors are country-clustered and finite-sample-corrected. Abbreviations: CI = confidence interval; CT = computed tomography; EVT = endovascular thrombectomy; IVT = intravenous thrombolysis; MIR = mortality-to-incidence ratio; SD = standard deviation; SDI = Socio-Demographic Index; SU = stroke unit; THE = total health expenditure; UHC = Universal Health Coverage.

### Sensitivity to the outcome definition, to incidence and to model form

Considering MIR separately (Table S15), we found no evidence of the positive between-country EVT–incidence association this mechanism would require; the incidence estimate was negative but imprecise (β_between = −0.24, 95% CI −0.67 to +0.19, *P* = .27). The between-country association was present when the death rate was analysed separately (−0.41, −0.80 to −0.03, *P* = .038; *P* = .046 in the four-wave extension). Adjustment for incidence nevertheless attenuated the MIR estimate to −0.33 (−0.67 to +0.02, *P* = .061); no within-country coefficient was inverse. The estimate was unchanged when SDI and age65+ were decomposed like EVT, or a country random intercept added (−0.42 in all three forms; Table S16), and larger without SDI adjustment (raw β_between = −0.020 against −0.013; Table S6).

### All-stroke endpoints and supporting specifications

All-stroke endpoint results were directionally consistent with the ischaemic-specific findings (Supplementary Material). The pooled OLS EVT coefficient was negative, as were the between-country coefficients of confounder-set variants S1–S7 (Tables S1 and S6). Influence diagnostics show how far the primary *P* value rests on single countries: Cook’s distance flagged Serbia, Albania, Georgia and Lithuania (Table S11), and in leave-one-country-out refits the between-country coefficient ranged from −0.29 to −0.51 SD with *P* from .007 to .061; 38 of 39 intervals excluded zero, the exception being the model without Portugal, and removing Serbia moved the estimate furthest towards the null (Table S12, Figure S1). The point estimate is stable in direction and magnitude; its conventional significance is not robust to the removal of a single country. Two-way fixed-effects and first-difference models identify only within-country associations. Their estimates provided no evidence of the hypothesised inverse association; most were compatible with the null, although the exploratory 2019–2020 first-difference estimate was positive (Table S3).

## Discussion

In this repeated cross-sectional ecological analysis of 39 European and adjacent countries, higher country-mean EVT activity in the source-homogeneous 2016, 2019 and 2020 panel was associated with lower GBD-derived ischaemic stroke MIR between countries (fully standardised β_between = −0.42, 95% CI −0.79 to −0.05), and no within-country association was detected once shared temporal trends were absorbed. An extension including 2023 data gave a similar estimate but is supportive only, given the source change and incomplete reporting. These findings are consistent with national EVT activity marking cross-national differences in organised acute stroke-service capacity. However, the between-country estimate they rest on is also the one most exposed to unmeasured national differences, and nothing here shows that increasing EVT provision reduces population stroke burden.

The findings extend two strands of evidence. The GBD analyses have documented marked international differences in stroke mortality and disability, and emphasised prevention, acute care and rehabilitation as components of burden reduction,^2^ and the World Stroke Organisation–Lancet Neurology Commission has called for national monitoring of stroke services.^20^ Previous surveys of European acute stroke care and global reperfusion availability described access to and delivery of treatment rather than repeated country-level associations with population burden.^9^^,^^10^^,^^16^ A Stroke Service Tracker report on 2023 data from 47 European nations documented wide variation in thrombectomy, stroke unit access and early mortality;^21^ because our 2023 extension draws on the same reporting infrastructure, that report contextualises rather than validates it. A companion analysis of the same 2023 Tracker data found EVT treatment proportions of 0.2% to 14.3% of ischaemic strokes, with no clear association with the density of EVT-capable centres, national stroke plans or quality programmes, suggesting that structural indicators alone do not explain cross-country variation in access.^22^ That analysis addresses infrastructure rather than delivered activity, so it does not test the association reported here; read alongside it, our finding is most consistent with EVT activity tracking whether the pathway functions rather than whether its components exist. Recent European data also show that greater neurointerventionalist density is associated with higher EVT delivery,^23^ supporting workforce availability as one component of delivered treatment capacity; harmonised workforce data were not available for our panel years and could not enter as a comparator, and neurointerventionalist density would be an informative covariate in a prospective evaluation. Our study adds an outcome-focused analysis across repeated waves and shows that the association with MIR is principally between countries.

An interpretation of EVT activity as a systems marker is organisationally plausible: delivering EVT at population level requires rapid recognition and transport, imaging and vascular diagnosis, transfer pathways, trained multidisciplinary teams, procedural availability, stroke unit care and post-acute support,^11^^,^^20^ so a national EVT rate may capture the functioning of several interdependent services rather than procedural capacity alone. The attenuation of the EVT–MIR estimate under adjustment for total health expenditure and UHC cuts against it, however. Between countries, national wealth and health-system resourcing move with both exposure and outcome, and attenuation under resource adjustment is the pattern expected if those resources confound part of the association. If EVT activity largely stands in for health-system resources, what survives adjustment is a between-country association of the same sign, about two-thirds of the primary estimate, the CI for which includes the null; these data cannot separate a stroke-service signal from a resource gradient, and we do not claim that they do. Among the acute-care indicators available for these countries, delivered EVT activity is nevertheless the one for which between-country gradient aligns with ischaemic stroke mortality relative to incidence. The IVT rate, CT scanner density and stroke unit density showed no comparable association. That does not make EVT a superior or unique measure: those data had different coverage and measurement structures, and the coefficients were not formally compared.

The direction of the between-country estimates was similar for YLLs and DALYs, whereas no association was detected for YLDs. This pattern should be interpreted cautiously: patient-level trials establish that EVT improves functional outcomes in eligible patients,^4–7^ whereas GBD-derived YLDs reflect prevalent disability accumulated across survivors and years, and a GBD 2023 analysis of European ischaemic stroke describes the same divergence at the level of burden itself, with YLLs falling about four times faster than YLDs.^24^ The analysis was not designed to compare endpoint-specific effects, and the YLD null does inform about whether EVT affects disability. The within-country term is less vulnerable to confounding by fixed national characteristics, since these drop out of it by design and it shows no inverse association: country-specific increases in EVT activity were not followed by reductions in MIR beyond common temporal trends. Within-country variation was, however, substantially smaller—about half the between-country variation in EVT and about one-sixth in MIR—making this estimate less precise; the design could detect only a within-country effect of at least 0.23 SD at 80% power. The null may therefore reflect limited temporal variation, measurement limitations or a genuine distinction between long-standing system capacity and short-term changes in procedure rates. The more precise between-country estimate remains the one more exposed to residual confounding, which is why we do not read it as evidence that the marker tracks change.

This study has limitations. First, the ecological design is vulnerable to residual confounding and cannot identify the causal effect of EVT on individual or population outcomes; countries with higher EVT activity might also differ in prevention, case mix, stroke severity, ascertainment and other aspects of care not captured at country level. The MIR is constructed from two modelled GBD rates, not observed case fatality, and GBD uncertainty was not propagated into the models. The GBD mortality and incidence models also use covariates related to sociodemographic development, so associations may partly reflect shared model inputs; the association was larger without SDI adjustment and was also present for the death rate alone, suggesting that it was not created simply by including SDI as a model covariate, but a contribution from shared inputs cannot be excluded. Conditioning on the incidence rate attenuated the estimate to the edge of conventional significance, and with only 39 countries, conventional statistical significance was sensitive to single-country deletion. Second, national EVT activity is an imperfect proxy for service capacity, and not the measure European monitoring uses: the Stroke Action Plan for Europe sets its thrombectomy target as a percentage of ischaemic strokes treated, whereas our exposure is procedures per head of population and so also reflects incidence and case mix,^11^ so this marker complements that indicator rather than replacing it, and a prospective test should use the penetration denominator. Within-country changes are further complicated by the extension of thrombectomy eligibility after DAWN (DWI or CTP Assessment with Clinical Mismatch in the Triage of Wake-Up and Late Presenting Strokes Undergoing Neurointervention with Trevo) and DEFUSE-3 (Endovascular Therapy Following Imaging Evaluation for Ischemic Stroke),^25^^,^^26^ so rising activity between 2016 and 2020 might partly reflect late-window selection within existing networks. Third, the 2023 extension is limited by a source transition and selective reporting, which is why the single-source three-wave panel is primary. Fourth, the comparator measures were heterogeneous and incompletely longitudinal, and adjustment for correlated macro-system indicators cannot fully separate stroke-service organisation from national resources. Fifth, the window includes 2020, when COVID-19 disrupted acute stroke pathways, although analyses excluding 2020 gave a compatible pattern. Finally, the sample comprised predominantly European, relatively high-income countries, so generalisability to settings with lower reperfusion availability or less complete surveillance is uncertain.

## Conclusion

National EVT activity was associated with lower GBD-derived ischaemic stroke MIR between countries in a source-homogeneous European panel, but the association attenuated after adjustment for health-system resources and stroke incidence, and no inverse within-country association was detected over 2016–2020. The EVT activity therefore remains a candidate marker for cross-country comparison rather than an established indicator for monitoring change, and should be interpreted alongside directly measured quality-of-care indicators. The Stroke Service Tracker provides an infrastructure for prospective evaluation using harmonised service metrics and directly observed outcomes.

## Supplementary Material

Supplementary_materials_aakag121

## Data Availability

This ecological study used aggregate country-year data and did not include individual participant data. The analysis code for all reported statistical analyses, tables and figures, together with a data dictionary describing the derived country-year variables, is publicly available in Mendeley Data (DOI: 10.17632/r9p47jkz8w.1) under an MIT licence, without access restrictions. No derived analytic datasets are deposited; the underlying country-year data must be reassembled from their original aggregate sources to rerun the analyses. Global Burden of Disease estimates are publicly available through the GBD Results Tool, and the remaining covariate sources are openly available from their respective publishers; the European Stroke Organisation Stroke Service Tracker–derived 2023 country-year data are not publicly available and can be requested, with permission, from the European Stroke Organisation. Full reproduction of the analyses therefore requires obtaining the European Stroke Organisation data under that permission. The study protocol, STROBE checklist and GATHER checklist are provided as supplementary files. No additional unpublished analytic data are available.

## References

[1] Global Burden of Disease Collaborative Network . Global Burden of Disease study 2023 (GBD 2023) results [Internet]. Seattle, United States: Institute for Health Metrics and Evaluation (IHME); 2024. Available from: https://vizhub.healthdata.org/gbd-results/ (last accessed date: 01 June 2026).

[2] GBD 2021 Stroke Risk Factor Collaborators . Global, regional, and national burden of stroke and its risk factors, 1990–2021: a systematic analysis for the global burden of disease study 2021. Lancet Neurol. 2024;23:973–1003. 10.1016/S1474-4422(24)00369-7 PMC12254192 39304265

[3] Feigin VL, Brainin M, Norrving B, et al. World stroke organization: global stroke fact sheet 2025. Int J Stroke. 2025;20:132–144. 10.1177/17474930241308142 PMC11786524 39635884

[4] Berkhemer OA, Fransen PSS, Beumer D, et al. A randomized trial of intraarterial treatment for acute ischemic stroke. N Engl J Med. 2015;372:11–20. 10.1056/NEJMoa1411587 25517348

[5] Goyal M, Demchuk AM, Menon BK, et al. Randomized assessment of rapid endovascular treatment of ischemic stroke. N Engl J Med. 2015;372:1019–1030. 10.1056/NEJMoa1414905 25671798

[6] Campbell BCV, Mitchell PJ, Kleinig TJ, et al. Endovascular therapy for ischemic stroke with perfusion-imaging selection. N Engl J Med. 2015;372:1009–1018. 10.1056/NEJMoa1414792 25671797

[7] Saver JL, Goyal M, Bonafe A, et al. Stent-retriever thrombectomy after intravenous t-PA vs. t-PA alone in stroke. N Engl J Med. 2015;372:2285–2295. 10.1056/NEJMoa1415061 25882376

[8] Turc G, Bhogal P, Fischer U, et al. European stroke organisation (ESO) – European Society for Minimally Invasive Neurological Therapy (ESMINT) guidelines on mechanical thrombectomy in acute ischaemic stroke. Eur Stroke J. 2019;4:6–12. 10.1177/2396987319832140 PMC6533858 31165090

[9] Aguiar de Sousa D, Wilkie A, Norrving B, et al. Delivery of acute ischaemic stroke treatments in the European region in 2019 and 2020. Eur Stroke J. 2023;8:618–628. 10.1177/23969873231186042 PMC10472963 37431768

[10] Kim J, Olaiya MT, De Silva DA, et al. Global stroke statistics 2023: availability of reperfusion services around the world. Int J Stroke. 2024;19:253–270. 10.1177/17474930231210448 PMC10903148 37853529

[11] Christensen H, Pezzella FR, Roaldsen MB, et al. Stroke Action Plan for Europe 2018–2030 (SAP-E): mid-term review and update. Eur Stroke J. 2026;11:aakaf026. 10.1093/esj/aakaf026 PMC12866651 41614539

[12] World Bank . World development indicators [Internet]. Washington (DC): The World Bank; 2024. Available from: https://databank.worldbank.org/source/world-development-indicators

[13] World Health Organization . Global health observatory data repository: Medical equipment [Internet]. Geneva: World Health Organization; 2024 [cited 2026 Mar 30]. Available from: https://www.who.int/data/gho

[14] von Elm E, Altman DG, Egger M, Pocock SJ, Gøtzsche PC, Vandenbroucke JP. The Strengthening the Reporting of Observational Studies in Epidemiology (STROBE) statement: guidelines for reporting observational studies. Lancet. 2007;370: 1453–1457. 10.1016/S0140-6736(07)61602-X 18064739

[15] Stevens GA, Alkema L, Black RE, et al. Guidelines for accurate and transparent health estimates reporting: the GATHER statement. Lancet. 2016;388:e19–e23. 10.1016/S0140-6736(16)30388-9 27371184

[16] Aguiar de Sousa D, von Martial R, Abilleira S, et al. Access to and delivery of acute ischaemic stroke treatments: a survey of national scientific societies and stroke experts in 44 European countries. Eur Stroke J. 2019;4:13–28. 10.1177/2396987318786023 PMC6533860 31165091

[17] European Stroke Organisation . Stroke action plan for Europe: Stroke service tracker [Internet]. European Stroke Organisation; 2025. Available from: https://actionplan.eso-stroke.org

[18] International Atomic Energy Agency . IMAGINE: Imaging Availability, Needs, and Equity database [Internet]. Vienna: International Atomic Energy Agency; 2022 [cited 2026 Mar 30]. Available from: https://humanhealth.iaea.org/HHW/DBStatistics/IMAGINE.html

[19] Organisation for Economic Co-operation and Development . OECD health statistics 2024 [Internet]. Paris: OECD Publishing; 2024 [cited 2026 Mar 30]. Available from: https://www.oecd.org/health/health-data.htm

[20] Feigin VL, Owolabi MO, World Stroke Organization–Lancet Neurology Commission Stroke Collaboration Group. Pragmatic solutions to reduce the global burden of stroke: a world stroke organization–lancet neurology commission. Lancet Neurol. 2023;22:1160–1206. 10.1016/S1474-4422(23)00277-6 PMC10715732 37827183

[21] Christensen H, Pezzella FR, Roaldsen MB, et al. Status on stroke and stroke care in Europe 2023: stroke service tracker 2023 data based on 1,460,360 strokes in 47 European nations. Eur Stroke J. 2026;11:aakag008. 10.1093/esj/aakag008 PMC12919429 41712681

[22] Wassvik L, Ovesen C, Tomek A, et al. Accessibility and use of organised stroke care and reperfusion therapies in Europe: results from the SAP-E stroke service tracker 2023. Eur Stroke J. 2026;11:aakag083. 10.1093/esj/aakag083 PMC13387431 42479897

[23] Mujanovic A, Romoli M, Sferruzza G, et al. Gaps in the access to endovascular thrombectomy for acute ischaemic stroke: estimating neurointerventional training needs and modelling implementation impact based on current thrombectomy indicators. Eur Stroke J. 2026;11:aakag026. 10.1093/esj/aakag026 PMC13171038 42127973

[24] Brigo F, Zini A, Groppa S, Winter Y, Lochner P. Trends in mortality and disability from ischaemic stroke in Europe, 1990–2023. Eur Stroke J. 2026;11:aakag082. 10.1093/esj/aakag082 PMC13387428 42479895

[25] Nogueira RG, Jadhav AP, Haussen DC, et al. Thrombectomy 6 to 24 hours after stroke with a mismatch between deficit and infarct. N Engl J Med. 2018;378:11–21. 10.1056/NEJMoa1706442 29129157

[26] Albers GW, Marks MP, Kemp S, et al. Thrombectomy for stroke at 6 to 16 hours with selection by perfusion imaging. N Engl J Med. 2018;378:708–718. 10.1056/NEJMoa1713973 PMC6590673 29364767

