# Supplementary material for: Endovascular thrombectomy activity as a candidate marker of organised acute stroke-service capacity: a global burden of disease-based European panel study
Source: Eur Stroke J. 2026 Oct 15;11(10):aakag121. doi: 10.1093/esj/aakag121 (PMC13671229; doi:10.1093/esj/aakag121)
Supplement: Supplementary file 1 [file supplementary_materials_aakag121.zip › supplementary_material-10.docx]

Supplementary Material

Endovascular thrombectomy activity as a candidate marker of organised acute stroke-service capacity: a Global Burden of Disease-based European panel study

This appendix contains additional tables and figures supporting the main analysis. The primary panel is the balanced three-wave Aguiar specification (39 countries, 2016/2019/2020; 117 country-year observations); the 4-wave extension adds the 2023 ESO Stroke Service Tracker wave (144 country-year observations, 27 of 39 countries reporting in 2023). Within–between models, pooled OLS, two-way fixed effects, comparator-exposure models, and the alternative-endpoint analysis all use country-clustered robust standard errors with finite-sample corrections; the first-difference models (Table S3) use HC1 heteroscedasticity-consistent standard errors instead, because at most one differenced observation enters per country in each wave-pair so cluster adjustment is not informative there. Eight stroke burden endpoints are reported (all-stroke MIR, YLLs, YLDs, DALYs and the equivalent ischaemic stroke endpoints).

## Supplementary Methods

**Precision of the within-country estimate.** Across all 117 country-year observations the standard deviation of EVT activity was 5.25 procedures per 100 000 between countries and 2.85 within them; for ischaemic stroke MIR the corresponding values were 0.179 and 0.030. The 80% power threshold for the within-country coefficient was calculated for a two-sided α = 0.05 test using the cluster-robust standard error and a *t* distribution with 38 degrees of freedom, and equals 0.23 SD of the outcome.

### EVT rate estimation for the 2023 wave

Wave-4 (2023) EVT rates were derived from the ESO Stroke Service Tracker, which reports mechanical thrombectomy (MT) as a percentage of ischaemic stroke admissions. We converted these to procedures per 100 000 population as (*p*/100) × (*a*/*N*) × 100 000, where *p* is the reported MT percentage, *a* the ischaemic stroke admissions, and *N* the total population, taking admissions and population from the same source for internal consistency. Two quality filters were applied. (1) Country-years with a missing or zero ischaemic stroke admission count were dropped, as the rate is then undefined; this excluded nine countries (Albania, Bosnia and Herzegovina, France, Georgia, Greece, Luxembourg, Montenegro, Serbia, and Slovenia). (2) An MT percentage of zero was prespecified to be treated as missing rather than as a true zero where the same country had recorded an Aguiar EVT rate of at least 1 per 100 000 in 2020, on the grounds that such a value reflects a reporting gap rather than genuine cessation of activity, and to be retained as a genuine zero in countries without prior recorded activity. In the event, no country reported a zero MT percentage for 2023: the rule therefore reclassified nothing, and this filter excluded only the three countries for which the Tracker carries no MT percentage at all (Croatia, Ireland, and Malta). United Kingdom constituent nations were aggregated to a single GBR value using admission-weighted percentages. Valid 2023 EVT values were accordingly available for 27 of the 39 panel countries, and every wave-4 exclusion rests on missing data rather than on a reported value. The Tracker provides country-level admission counts and MT percentages reported by national coordinators. Table S17 sets each reporter’s stated registry coverage and its admission count against the GBD 2023 incident case count: coverage was reported as complete by 15 of the 27 countries, partial (11–97%) by 8, and not reported by 4, and admissions ranged from 0.36 to 1.91 times the GBD incident count (median 1.23; 26 countries with a GBD 2023 estimate). Where registry coverage was incomplete, deriving a population rate assumes that the reported MT percentage is representative of the reported national admission count. We therefore treat the 2023 rates as country-level estimates rather than fully ascertained national rates in those countries.

### Additional data-source details

CT scanner density was compiled from the IAEA IMAGINE database, OECD Health Statistics, and the WHO Global Health Observatory, taken in that priority order, with linear interpolation between observed datapoints where intermediate years were missing (per-country audit in Table S10). The comparator and adjustment covariates — IVT rate, stroke unit density, total health expenditure per capita, UHC service coverage, and the Gini index — were assembled from ESO surveys, the WHO Global Health Expenditure Database, WHO/World Bank UHC tracking, and the World Bank Development Indicators. The Aguiar surveys report IVT for 2019 and 2020 separately; the two were averaged into one country-level value applied to both waves, so IVT carries no within-country variation in the primary panel and enters the comparator models as a between-country term. IVT for 2023 was computed from the Stroke Service Tracker analogously to EVT, inheriting the same Aguiar-to-Tracker source change between waves 3 and 4; stroke unit density was available only as a country-level snapshot without wave-specific temporal variation. The Socio-Demographic Index (GBD 2023 release) and the proportion of the population aged 65 years or older (World Bank Development Indicators) were the adjustment variables in all primary models.

### Supporting specifications and diagnostics

Beyond the primary within–between model, we report pooled OLS (Table S1); two-way (country and wave) fixed effects (Table S2); first-difference models (Table S3); a fixed-effects specification excluding the 2020 (COVID-19) wave (Table S4); cross-sectional OLS by year (Table S5); and confounder-set variants S1–S7 (+ Gini; + IVT + stroke units; UHC-only; THE-only; population-weighted; age 65+ only, without SDI; wave fixed effects only; Table S6). Three further tables were added at revision: EVT against the incidence and death rates that make up MIR, and against MIR conditional on incidence (Table S15); the primary model refitted with SDI and age 65+ decomposed into country means and deviations, and as a correlated random-effects model with a country random intercept (Table S16); and the completeness of the 2023 Tracker admission counts (Table S17). Influence was assessed with Cook’s distance (Table S11) and leave-one-country-out estimation (Table S12, Figure S1). All models use country-clustered robust standard errors with finite-sample corrections, except the first-difference models, which use HC1 heteroscedasticity-consistent standard errors because at most one differenced observation enters per country in each wave-pair, making cluster adjustment uninformative.

### Protocol amendments

The study was prespecified with the unbalanced four-wave within–between analysis (2016, 2019, 2020, 2023; 144 country-year observations) as the primary model; the balanced three-wave Aguiar specification (2016/2019/2020) and a binary source indicator (Aguiar versus Stroke Service Tracker) were both listed as prespecified source-change sensitivities. After data assembly but before inspection of any outcome regression results, two amendments were made.

First, the primary specification and the Aguiar-only three-wave sensitivity were exchanged: the balanced three-wave Aguiar panel became the primary model because the wave-3-to-4 data-source change and the unbalanced wave-4 reporting (27 of 39 countries) compromised the between-country EVT mean as a uniform cross-country construct. The four-wave specification is retained and centrally reported as a secondary extension.

Second, the prespecified source-indicator analysis became collinear with the 2023 wave indicator in the four-wave specification with wave fixed effects and was therefore no longer separately identifiable; the three source-change sensitivities reported in this appendix address source change instead.

No outcome model results had been inspected when these amendments were made, and both are logged in the repository commit history.

## Supplementary Tables

Table S1. Pooled OLS regression results

| Endpoint | Covariate | β | SE | p | 95% CI | n |
| --- | --- | --- | --- | --- | --- | --- |
| All-stroke MIR | EVT rate | -0.0099 | 0.0039 | 0.015 | [-0.0178, -0.0021] | 117 |
| All-stroke MIR | SDI | -1.7594 | 0.2844 | <0.0001 | [-2.3352, -1.1837] | 117 |
| All-stroke MIR | % aged 65+ | 0.0124 | 0.0075 | 0.11 | [-0.0028, 0.0275] | 117 |
| All-stroke YLLs | EVT rate | -73.9489 | 32.0078 | 0.026 | [-138.7452, -9.1525] | 117 |
| All-stroke YLLs | SDI | -12702.2461 | 2206.6729 | <0.0001 | [-17169.4218, -8235.0704] | 117 |
| All-stroke YLLs | % aged 65+ | 117.5824 | 65.4430 | 0.080 | [-14.8999, 250.0648] | 117 |
| All-stroke YLDs | EVT rate | -0.6412 | 2.1487 | 0.77 | [-4.9911, 3.7087] | 117 |
| All-stroke YLDs | SDI | -472.0933 | 174.6540 | 0.010 | [-825.6618, -118.5249] | 117 |
| All-stroke YLDs | % aged 65+ | 15.6482 | 4.7000 | 0.0019 | [6.1336, 25.1628] | 117 |
| All-stroke DALYs | EVT rate | -74.5901 | 33.8131 | 0.034 | [-143.0412, -6.1390] | 117 |
| All-stroke DALYs | SDI | -13174.3394 | 2300.6867 | <0.0001 | [-17831.8361, -8516.8426] | 117 |
| All-stroke DALYs | % aged 65+ | 133.2306 | 69.0992 | 0.061 | [-6.6534, 273.1146] | 117 |
| Ischaemic stroke MIR | EVT rate | -0.0094 | 0.0042 | 0.032 | [-0.0179, -0.0008] | 117 |
| Ischaemic stroke MIR | SDI | -1.4263 | 0.3770 | 0.00053 | [-2.1895, -0.6631] | 117 |
| Ischaemic stroke MIR | % aged 65+ | 0.0176 | 0.0084 | 0.043 | [0.0006, 0.0346] | 117 |
| Ischaemic stroke YLLs | EVT rate | -49.0468 | 25.4925 | 0.062 | [-100.6538, 2.5601] | 117 |
| Ischaemic stroke YLLs | SDI | -7896.3869 | 1900.0023 | 0.00018 | [-11742.7404, -4050.0334] | 117 |
| Ischaemic stroke YLLs | % aged 65+ | 110.6220 | 52.1423 | 0.040 | [5.0653, 216.1786] | 117 |
| Ischaemic stroke YLDs | EVT rate | -0.1344 | 1.9714 | 0.95 | [-4.1253, 3.8565] | 117 |
| Ischaemic stroke YLDs | SDI | -520.5452 | 154.2053 | 0.0017 | [-832.7175, -208.3729] | 117 |
| Ischaemic stroke YLDs | % aged 65+ | 11.7915 | 4.0828 | 0.0064 | [3.5263, 20.0568] | 117 |
| Ischaemic stroke DALYs | EVT rate | -49.1812 | 27.1857 | 0.078 | [-104.2159, 5.8534] | 117 |
| Ischaemic stroke DALYs | SDI | -8416.9320 | 2016.3627 | 0.00017 | [-12498.8448, -4335.0192] | 117 |
| Ischaemic stroke DALYs | % aged 65+ | 122.4135 | 55.7500 | 0.034 | [9.5535, 235.2735] | 117 |

CI indicates confidence interval; DALYs, disability-adjusted life-years; EVT, endovascular thrombectomy; MIR, mortality-to-incidence ratio; OLS, ordinary least squares; SDI, Socio-Demographic Index; SE, standard error; YLDs, years lived with disability; YLLs, years of life lost.

Table S2. Two-way fixed effects regression results

| Endpoint | Covariate | β | SE | p | 95% CI | n |
| --- | --- | --- | --- | --- | --- | --- |
| All-stroke MIR | EVT rate | 0.0024 | 0.0024 | 0.32 | [-0.0024, 0.0071] | 117 |
| All-stroke MIR | SDI | 1.4731 | 4.2825 | 0.73 | [-7.0620, 10.0081] | 117 |
| All-stroke MIR | % aged 65+ | -0.0152 | 0.0213 | 0.48 | [-0.0576, 0.0271] | 117 |
| All-stroke YLLs | EVT rate | -0.9835 | 9.9957 | 0.92 | [-20.9049, 18.9379] | 117 |
| All-stroke YLLs | SDI | 6161.1620 | 18227.6837 | 0.74 | [-30166.5593, 42488.8833] | 117 |
| All-stroke YLLs | % aged 65+ | -94.7712 | 85.2322 | 0.27 | [-264.6388, 75.0964] | 117 |
| All-stroke YLDs | EVT rate | -0.0261 | 0.3447 | 0.94 | [-0.7131, 0.6609] | 117 |
| All-stroke YLDs | SDI | -947.8122 | 689.5192 | 0.17 | [-2322.0219, 426.3976] | 117 |
| All-stroke YLDs | % aged 65+ | 7.8828 | 3.4578 | 0.026 | [0.9914, 14.7742] | 117 |
| All-stroke DALYs | EVT rate | -1.0096 | 9.9368 | 0.92 | [-20.8135, 18.7944] | 117 |
| All-stroke DALYs | SDI | 5213.3498 | 17689.8123 | 0.77 | [-30042.3953, 40469.0949] | 117 |
| All-stroke DALYs | % aged 65+ | -86.8884 | 84.1647 | 0.31 | [-254.6283, 80.8516] | 117 |
| Ischaemic stroke MIR | EVT rate | 0.0031 | 0.0023 | 0.19 | [-0.0016, 0.0077] | 117 |
| Ischaemic stroke MIR | SDI | 0.6300 | 4.2924 | 0.88 | [-7.9247, 9.1848] | 117 |
| Ischaemic stroke MIR | % aged 65+ | -0.0086 | 0.0208 | 0.68 | [-0.0500, 0.0328] | 117 |
| Ischaemic stroke YLLs | EVT rate | 0.1339 | 7.2830 | 0.99 | [-14.3810, 14.6488] | 117 |
| Ischaemic stroke YLLs | SDI | 2303.2998 | 11665.9285 | 0.84 | [-20946.8623, 25553.4618] | 117 |
| Ischaemic stroke YLLs | % aged 65+ | -48.2824 | 56.1980 | 0.39 | [-160.2849, 63.7201] | 117 |
| Ischaemic stroke YLDs | EVT rate | -0.0903 | 0.2179 | 0.68 | [-0.5246, 0.3441] | 117 |
| Ischaemic stroke YLDs | SDI | -96.3902 | 205.8417 | 0.64 | [-506.6320, 313.8517] | 117 |
| Ischaemic stroke YLDs | % aged 65+ | 7.1006 | 2.0528 | 0.00091 | [3.0094, 11.1917] | 117 |
| Ischaemic stroke DALYs | EVT rate | 0.0436 | 7.3569 | 0.99 | [-14.6187, 14.7059] | 117 |
| Ischaemic stroke DALYs | SDI | 2206.9096 | 11708.3982 | 0.85 | [-21127.8944, 25541.7135] | 117 |
| Ischaemic stroke DALYs | % aged 65+ | -41.1818 | 56.8642 | 0.47 | [-154.5120, 72.1484] | 117 |

CI indicates confidence interval; DALYs, disability-adjusted life-years; EVT, endovascular thrombectomy; MIR, mortality-to-incidence ratio; SDI, Socio-Demographic Index; SE, standard error; YLDs, years lived with disability; YLLs, years of life lost.

Table S3. First-difference regression results

| Endpoint | Period | EVT β | EVT p | EVT 95% CI | n |
| --- | --- | --- | --- | --- | --- |
| All-stroke MIR | 2016 → 2019 | 0.0014 | 0.47 | [-0.0025, 0.0053] | 39 |
| All-stroke MIR | 2019 → 2020 | 0.0042 | 0.012 | [0.0009, 0.0075] | 39 |
| All-stroke MIR | 2016 → 2020 | 0.0030 | 0.22 | [-0.0018, 0.0079] | 39 |
| All-stroke YLLs | 2016 → 2019 | -3.3744 | 0.70 | [-20.2630, 13.5141] | 39 |
| All-stroke YLLs | 2019 → 2020 | 7.9466 | 0.28 | [-6.5905, 22.4837] | 39 |
| All-stroke YLLs | 2016 → 2020 | -1.3692 | 0.89 | [-21.1082, 18.3698] | 39 |
| All-stroke YLDs | 2016 → 2019 | -0.0144 | 0.96 | [-0.5805, 0.5516] | 39 |
| All-stroke YLDs | 2019 → 2020 | -0.5976 | 0.12 | [-1.3553, 0.1600] | 39 |
| All-stroke YLDs | 2016 → 2020 | -0.0108 | 0.98 | [-0.7225, 0.7009] | 39 |
| All-stroke DALYs | 2016 → 2019 | -3.3889 | 0.69 | [-20.2948, 13.5170] | 39 |
| All-stroke DALYs | 2019 → 2020 | 7.3490 | 0.33 | [-7.4904, 22.1883] | 39 |
| All-stroke DALYs | 2016 → 2020 | -1.3800 | 0.89 | [-20.9046, 18.1446] | 39 |
| Ischaemic stroke MIR | 2016 → 2019 | 0.0022 | 0.25 | [-0.0015, 0.0059] | 39 |
| Ischaemic stroke MIR | 2019 → 2020 | 0.0040 | 0.014 | [0.0008, 0.0072] | 39 |
| Ischaemic stroke MIR | 2016 → 2020 | 0.0036 | 0.14 | [-0.0011, 0.0083] | 39 |
| Ischaemic stroke YLLs | 2016 → 2019 | -1.4988 | 0.82 | [-14.1499, 11.1523] | 39 |
| Ischaemic stroke YLLs | 2019 → 2020 | 4.5562 | 0.35 | [-5.0055, 14.1179] | 39 |
| Ischaemic stroke YLLs | 2016 → 2020 | -0.3729 | 0.96 | [-14.5123, 13.7666] | 39 |
| Ischaemic stroke YLDs | 2016 → 2019 | -0.0133 | 0.95 | [-0.3952, 0.3686] | 39 |
| Ischaemic stroke YLDs | 2019 → 2020 | -0.5031 | 0.18 | [-1.2372, 0.2310] | 39 |
| Ischaemic stroke YLDs | 2016 → 2020 | -0.1408 | 0.50 | [-0.5481, 0.2665] | 39 |
| Ischaemic stroke DALYs | 2016 → 2019 | -1.5121 | 0.82 | [-14.3179, 11.2938] | 39 |
| Ischaemic stroke DALYs | 2019 → 2020 | 4.0531 | 0.43 | [-5.9919, 14.0981] | 39 |
| Ischaemic stroke DALYs | 2016 → 2020 | -0.5137 | 0.94 | [-14.7740, 13.7466] | 39 |

CI indicates confidence interval; DALYs, disability-adjusted life-years; EVT, endovascular thrombectomy; MIR, mortality-to-incidence ratio; YLDs, years lived with disability; YLLs, years of life lost.

Table S4. Two-way fixed effects excluding 2020 (COVID sensitivity)

| Endpoint | Covariate | β | SE | p | 95% CI | n |
| --- | --- | --- | --- | --- | --- | --- |
| All-stroke MIR | EVT rate | 0.0014 | 0.0028 | 0.62 | [-0.0043, 0.0071] | 78 |
| All-stroke MIR | SDI | 1.7115 | 4.7639 | 0.72 | [-7.9598, 11.3828] | 78 |
| All-stroke MIR | % aged 65+ | -0.0134 | 0.0246 | 0.59 | [-0.0633, 0.0365] | 78 |
| All-stroke YLLs | EVT rate | -3.3744 | 12.1860 | 0.78 | [-28.1133, 21.3644] | 78 |
| All-stroke YLLs | SDI | 3517.1127 | 19708.8660 | 0.86 | [-36494.0124, 43528.2377] | 78 |
| All-stroke YLLs | % aged 65+ | -104.9395 | 103.3030 | 0.32 | [-314.6558, 104.7768] | 78 |
| All-stroke YLDs | EVT rate | -0.0144 | 0.4084 | 0.97 | [-0.8436, 0.8147] | 78 |
| All-stroke YLDs | SDI | -999.3156 | 710.7552 | 0.17 | [-2442.2254, 443.5941] | 78 |
| All-stroke YLDs | % aged 65+ | 5.7261 | 3.7215 | 0.13 | [-1.8291, 13.2812] | 78 |
| All-stroke DALYs | EVT rate | -3.3889 | 12.1985 | 0.78 | [-28.1531, 21.3753] | 78 |
| All-stroke DALYs | SDI | 2517.7970 | 19204.8587 | 0.90 | [-36470.1388, 41505.7329] | 78 |
| All-stroke DALYs | % aged 65+ | -99.2134 | 103.5941 | 0.34 | [-309.5206, 111.0937] | 78 |
| Ischaemic stroke MIR | EVT rate | 0.0022 | 0.0027 | 0.42 | [-0.0032, 0.0076] | 78 |
| Ischaemic stroke MIR | SDI | 0.4720 | 4.9475 | 0.92 | [-9.5720, 10.5159] | 78 |
| Ischaemic stroke MIR | % aged 65+ | -0.0090 | 0.0247 | 0.72 | [-0.0591, 0.0411] | 78 |
| Ischaemic stroke YLLs | EVT rate | -1.4988 | 9.1284 | 0.87 | [-20.0304, 17.0328] | 78 |
| Ischaemic stroke YLLs | SDI | -528.4258 | 13081.8471 | 0.97 | [-27085.9874, 26029.1357] | 78 |
| Ischaemic stroke YLLs | % aged 65+ | -61.4420 | 69.3291 | 0.38 | [-202.1876, 79.3036] | 78 |
| Ischaemic stroke YLDs | EVT rate | -0.0133 | 0.2755 | 0.96 | [-0.5727, 0.5461] | 78 |
| Ischaemic stroke YLDs | SDI | -206.2956 | 275.0198 | 0.46 | [-764.6154, 352.0242] | 78 |
| Ischaemic stroke YLDs | % aged 65+ | 5.6177 | 2.3689 | 0.023 | [0.8086, 10.4267] | 78 |
| Ischaemic stroke DALYs | EVT rate | -1.5121 | 9.2401 | 0.87 | [-20.2704, 17.2463] | 78 |
| Ischaemic stroke DALYs | SDI | -734.7214 | 13144.4941 | 0.96 | [-27419.4630, 25950.0202] | 78 |
| Ischaemic stroke DALYs | % aged 65+ | -55.8243 | 70.6149 | 0.43 | [-199.1802, 87.5316] | 78 |

CI indicates confidence interval; DALYs, disability-adjusted life-years; EVT, endovascular thrombectomy; MIR, mortality-to-incidence ratio; SDI, Socio-Demographic Index; SE, standard error; YLDs, years lived with disability; YLLs, years of life lost.

Table S5. Cross-sectional OLS by year (2016, 2019, 2020)

| Year | Endpoint | EVT β | EVT p | Adj R² | n |
| --- | --- | --- | --- | --- | --- |
| 2016 | All-stroke MIR | -0.0245 | 0.0018 | 0.539 | 39 |
| 2016 | All-stroke YLLs | -153.4555 | 0.014 | 0.464 | 39 |
| 2016 | All-stroke YLDs | -0.8257 | 0.85 | 0.210 | 39 |
| 2016 | All-stroke DALYs | -154.2812 | 0.018 | 0.452 | 39 |
| 2016 | Ischaemic stroke MIR | -0.0231 | 0.0070 | 0.387 | 39 |
| 2016 | Ischaemic stroke YLLs | -102.2233 | 0.034 | 0.350 | 39 |
| 2016 | Ischaemic stroke YLDs | -0.8846 | 0.80 | 0.223 | 39 |
| 2016 | Ischaemic stroke DALYs | -103.1079 | 0.043 | 0.343 | 39 |
| 2019 | All-stroke MIR | -0.0110 | 0.011 | 0.526 | 39 |
| 2019 | All-stroke YLLs | -86.1488 | 0.011 | 0.485 | 39 |
| 2019 | All-stroke YLDs | -0.6091 | 0.80 | 0.261 | 39 |
| 2019 | All-stroke DALYs | -86.7579 | 0.014 | 0.476 | 39 |
| 2019 | Ischaemic stroke MIR | -0.0103 | 0.032 | 0.376 | 39 |
| 2019 | Ischaemic stroke YLLs | -56.3768 | 0.034 | 0.365 | 39 |
| 2019 | Ischaemic stroke YLDs | -0.2998 | 0.88 | 0.266 | 39 |
| 2019 | Ischaemic stroke DALYs | -56.6765 | 0.044 | 0.360 | 39 |
| 2020 | All-stroke MIR | -0.0081 | 0.062 | 0.455 | 39 |
| 2020 | All-stroke YLLs | -72.4917 | 0.030 | 0.445 | 39 |
| 2020 | All-stroke YLDs | -0.6429 | 0.78 | 0.259 | 39 |
| 2020 | All-stroke DALYs | -73.1346 | 0.034 | 0.440 | 39 |
| 2020 | Ischaemic stroke MIR | -0.0073 | 0.13 | 0.322 | 39 |
| 2020 | Ischaemic stroke YLLs | -46.4869 | 0.074 | 0.334 | 39 |
| 2020 | Ischaemic stroke YLDs | -0.0911 | 0.96 | 0.269 | 39 |
| 2020 | Ischaemic stroke DALYs | -46.5779 | 0.090 | 0.331 | 39 |

DALYs indicates disability-adjusted life-years; EVT, endovascular thrombectomy; MIR, mortality-to-incidence ratio; OLS, ordinary least squares; YLDs, years lived with disability; YLLs, years of life lost.

Table S6. within–between sensitivity models S1–S7 for ischaemic stroke MIR

| Model | Description | n countries | n obs | EVT between β | EVT between p | EVT within p |
| --- | --- | --- | --- | --- | --- | --- |
| Primary | SDI + Age65+ | 39 | 117 | -0.0127 | 0.027 | 0.24 |
| S1: + Gini | SDI + Age65+ + Gini index | 37 | 110 | -0.0120 | 0.046 | 0.092 |
| S2: + IVT + SU | SDI + Age65+ + IVT rate + Stroke units | 36 | 72 | -0.0150 | 0.0056 | 0.054 |
| S3: UHC | UHC + Age65+ | 37 | 111 | -0.0092 | 0.054 | 0.12 |
| S4: THE | THE per capita + Age65+ | 37 | 111 | -0.0084 | 0.029 | 0.016 |
| S5: Pop-weighted | SDI + Age65+ (pop-weighted WLS) | 37 | 111 | -0.0142 | 0.027 | 0.39 |
| S6: no SDI | Age65+ only (no SDI) | 39 | 117 | -0.0201 | 0.00093 | 0.19 |
| S7: unadjusted | Wave fixed effects only (no covariates) | 39 | 117 | -0.0162 | 0.0025 | 0.19 |

EVT indicates endovascular thrombectomy; IVT, intravenous thrombolysis; MIR, mortality-to-incidence ratio; SDI, Socio-Demographic Index; THE, total health expenditure; UHC, Universal Health Coverage.

Table S7. Descriptive comparison of 27 countries with valid 2023 EVT data (reporters) vs 12 countries without (non-reporters), on mean pre-2023 (2016/2019/2020) values for EVT activity, ischaemic stroke MIR, Socio-Demographic Index, and proportion aged 65 years or older.

| Group | n countries | Mean EVT rate | Mean MIR | Mean SDI | Mean age ≥65 (%) |
| --- | --- | --- | --- | --- | --- |
| Reporters (n=27) | 27 | 8.21 | 0.477 | 0.817 | 18.43 |
| Non-reporters (n=12) | 12 | 4.12 | 0.595 | 0.791 | 17.85 |

EVT indicates endovascular thrombectomy; MIR, mortality-to-incidence ratio; SDI, Socio-Demographic Index.

Table S8. Alternative-endpoint sensitivity analysis using GBD-derived ischaemic stroke deaths rate (per 100,000) as the primary endpoint, replacing MIR. within–between EVT decomposition adjusted for SDI and age65+.

| Endpoint | n countries | n obs | Between β (95% CI) | Between p | Within β (95% CI) | Within p |
| --- | --- | --- | --- | --- | --- | --- |
| Ischaemic stroke deaths rate per 100,000 | 39 | 117 | -4.5298 [-8.7842, -0.2755] | 0.038 | -0.1101 [-1.2601, 1.0399] | 0.85 |

CI indicates confidence interval; EVT, endovascular thrombectomy; GBD, Global Burden of Disease; MIR, mortality-to-incidence ratio; SDI, Socio-Demographic Index.

Table S9. Per-variable per-wave missingness summary across the 39-country panel. Each cell shows the number of countries with a non-null value for that variable in that wave; the denominator is 39 (the panel size). Variables are grouped into panel-frozen (exposure, primary covariates, GBD outcomes) and master-table (alternative endpoint, sensitivity covariates). Notes document interpolation, exclusion criteria, and structural data constraints.

| Variable | 2016 | 2019 | 2020 | 2023 | Notes |
| --- | --- | --- | --- | --- | --- |
| EVT rate (per 100,000) | 39/39 | 39/39 | 39/39 | 27/39 | Aguiar surveys 2016/2019/2020; ESO Stroke Service Tracker 2023 (27 of 39 reporting). Zero-MT reports in countries with Aguiar wave-3 EVT >=1 treated as missing. |
| CT scanner density (per 100,000) | 39/39 | 39/39 | 39/39 | 23/39 | IAEA IMAGINE, OECD, WHO GHO; linearly interpolated between observed years; some countries extrapolated. See Table S10 (per-country CT interpolation audit), which reports the source values per million population; the analysis variable rescales them to per 100,000. |
| Ischaemic stroke YLLs / 100,000 | 39/39 | 39/39 | 39/39 | 27/39 | GBD 2023 release (ischaemic-specific extract); counted in panel_frozen.csv. The 2023 column reports availability within the analytic panel only — i.e. the 27 EVT-reporting countries, since panel_frozen.csv does not carry 2023 rows for the 12 non-reporters. |
| Ischaemic stroke DALYs / 100,000 | 39/39 | 39/39 | 39/39 | 27/39 | GBD 2023 release (ischaemic-specific extract); counted in panel_frozen.csv (same 2023 caveat as for ischaemic YLLs). |
| Ischaemic stroke YLDs / 100,000 | 39/39 | 39/39 | 39/39 | 27/39 | GBD 2023 release (ischaemic-specific extract); counted in panel_frozen.csv (same 2023 caveat as for ischaemic YLLs). |
| Socio-Demographic Index | 39/39 | 39/39 | 39/39 | 38/39 | GBD 2023 composite; counted in master_table for panel ISOs (independent of EVT panel inclusion). |
| Population aged >=65 (%) | 39/39 | 39/39 | 39/39 | 38/39 | World Bank Development Indicators; counted in master_table for panel ISOs. |
| Ischaemic stroke MIR (primary endpoint) | 39/39 | 39/39 | 39/39 | 38/39 | GBD 2023 release; counted in master_table for panel ISOs. |
| Ischaemic stroke deaths rate / 100,000 (alt endpoint) | 39/39 | 39/39 | 39/39 | 38/39 | GBD 2023 release; missing where master row absent (ARM, GEO partial). |
| IVT rate (per 100,000; sensitivity) | 0/39 | 36/39 | 36/39 | 26/39 | Aguiar 2023 for 2019/2020; ESO Stroke Service Tracker 2023 (same source change as EVT). No 2016 data. |
| Stroke unit density (per million; sensitivity) | 0/39 | 37/39 | 37/39 | 37/39 | ESO survey snapshot; one value per country, no within-country temporal variation. |
| THE per capita (USD; sensitivity) | 37/39 | 37/39 | 37/39 | 37/39 | WHO Global Health Expenditure Database; missing for ARM/GEO and a few small states. |
| UHC service coverage (0-100; sensitivity) | 37/39 | 37/39 | 37/39 | 37/39 | WHO/World Bank UHC tracking; missing for ARM/GEO and a few small states. |
| Gini index (0-100; sensitivity) | 37/39 | 37/39 | 36/39 | 32/39 | World Bank WDI; missing for ARM/GEO and a few small states. |
| Population (millions; S5 weights) | 37/39 | 37/39 | 37/39 | 37/39 | World Bank WDI; missing where master row absent. |

CT indicates computed tomography; DALYs, disability-adjusted life-years; ESO, European Stroke Organisation; EVT, endovascular thrombectomy; GBD, Global Burden of Disease; IVT, intravenous thrombolysis; MIR, mortality-to-incidence ratio; MT, mechanical thrombectomy; THE, total health expenditure; UHC, Universal Health Coverage; YLDs, years lived with disability; YLLs, years of life lost.

Table S10. CT scanner data: observed vs interpolated values by country, in scanners per million population. The analysis variable is these values rescaled to scanners per 100 000 population, as reported in Table 1 and Table S9

| Country (ISO3) | Status | 2016 | 2019 | 2020 | 2023 |
| --- | --- | --- | --- | --- | --- |
| ALB | Missing | 5.4 | 5.4 | 5.4 | – |
| ARM | Missing | 3.0 | 3.0 | 3.0 | – |
| AUT | Observed/Interpolated | 29.1 | 28.7 | 28.5 | 28.2 |
| BEL | Observed/Interpolated | 23.9 | 24.1 | 24.0 | 26.0 |
| BGR | Observed/Interpolated | 35.8 | 41.6 | 42.9 | 49.3 |
| BIH | Missing | 16.4 | 16.4 | 16.4 | – |
| CHE | Observed/Interpolated | 38.9 | 38.7 | 39.6 | 39.9 |
| CZE | Observed/Interpolated | 15.5 | 16.4 | 16.3 | 16.6 |
| DEU | Observed/Interpolated | 35.2 | 35.3 | 35.9 | 37.1 |
| DNK | Observed/Interpolated | 39.1 | 26.7 | 26.9 | 28.8 |
| ESP | Observed/Interpolated | 19.2 | 19.9 | 20.6 | 21.7 |
| EST | Observed/Interpolated | 17.5 | 18.8 | 20.3 | 21.9 |
| FIN | Observed/Interpolated | 24.2 | 16.3 | 17.0 | 18.1 |
| FRA | Observed/Interpolated | 16.9 | 18.1 | 18.9 | 21.0 |
| GBR | Observed/Interpolated | 9.5 | 9.0 | 8.9 | 9.9 |
| GEO | Missing | 8.8 | 8.8 | 8.8 | – |
| GRC | Observed/Interpolated | 35.9 | 42.5 | 43.7 | 50.6 |
| HRV | Observed/Interpolated | 18.0 | 20.5 | 23.0 | 23.6 |
| HUN | Observed/Interpolated | 8.9 | 9.5 | 9.7 | 11.2 |
| IRL | Observed/Interpolated | 17.2 | 21.1 | 20.0 | 19.2 |
| ISR | Observed/Interpolated | 9.5 | 9.7 | 9.6 | 10.0 |
| ITA | Observed/Interpolated | 34.6 | 36.5 | 37.4 | 41.5 |
| LTU | Observed/Interpolated | 22.9 | 26.3 | 31.0 | 33.1 |
| LUX | Observed/Interpolated | 17.2 | 16.1 | 22.2 | 25.5 |
| LVA | Observed/Interpolated | 36.2 | 37.1 | 37.4 | 40.5 |
| MDA | Missing | 5.5 | 5.5 | 5.5 | – |
| MKD | Missing | 6.2 | 6.2 | 6.2 | – |
| MLT | Observed/Interpolated | 9.3 | 9.3 | 9.3 | 16.3 |
| MNE | Missing | 16.1 | 16.1 | 16.1 | – |
| NLD | Observed/Interpolated | 13.0 | 14.9 | 14.7 | 16.6 |
| NOR | Observed/Interpolated | 15.5 | 26.4 | 31.0 | 28.1 |
| POL | Observed/Interpolated | 17.3 | 18.2 | 20.3 | 23.8 |
| PRT | Observed/Interpolated | 22.7 | 20.2 | 19.4 | 18.6 |
| ROU | Observed/Interpolated | 12.6 | 17.5 | 19.1 | 27.6 |
| SRB | Observed/Interpolated | 13.7 | 13.7 | 13.7 | 19.2 |
| SVK | Observed/Interpolated | 17.3 | 17.8 | 19.1 | 20.3 |
| SVN | Observed/Interpolated | 14.0 | 18.2 | 19.0 | 17.4 |
| SWE | Observed/Interpolated | 22.1 | 26.2 | 28.3 | 24.2 |
| UKR | Missing | 9.6 | 9.6 | 9.6 | – |

CT indicates computed tomography.

Table S11. Cook’s Distance for the within–between model (ischaemic stroke MIR)

| Country | ISO3 | Max Cook’s D | >4/n=0.0342 |
| --- | --- | --- | --- |
| Serbia | SRB | 0.0749 | Yes |
| Albania | ALB | 0.0544 | Yes |
| Georgia | GEO | 0.0459 | Yes |
| Lithuania | LTU | 0.0410 | Yes |
| Spain | ESP | 0.0198 | No |
| Latvia | LVA | 0.0170 | No |
| Armenia | ARM | 0.0170 | No |
| Israel | ISR | 0.0168 | No |
| Montenegro | MNE | 0.0151 | No |
| United Kingdom | GBR | 0.0138 | No |
| Moldova | MDA | 0.0138 | No |
| Austria | AUT | 0.0137 | No |
| Greece | GRC | 0.0132 | No |
| Sweden | SWE | 0.0115 | No |
| Switzerland | CHE | 0.0090 | No |
| Norway | NOR | 0.0086 | No |
| Ireland | IRL | 0.0065 | No |
| Estonia | EST | 0.0062 | No |
| Germany | DEU | 0.0053 | No |
| Slovenia | SVN | 0.0050 | No |
| Slovakia | SVK | 0.0044 | No |
| Luxembourg | LUX | 0.0039 | No |
| Finland | FIN | 0.0036 | No |
| France | FRA | 0.0029 | No |
| Bulgaria | BGR | 0.0028 | No |
| N. Macedonia | MKD | 0.0023 | No |
| Malta | MLT | 0.0022 | No |
| Ukraine | UKR | 0.0021 | No |
| Romania | ROU | 0.0019 | No |
| Bosnia & Herz. | BIH | 0.0018 | No |
| Italy | ITA | 0.0017 | No |
| Croatia | HRV | 0.0012 | No |
| Belgium | BEL | 0.0009 | No |
| Portugal | PRT | 0.0008 | No |
| Netherlands | NLD | 0.0007 | No |
| Denmark | DNK | 0.0005 | No |
| Czechia | CZE | 0.0005 | No |
| Poland | POL | 0.0004 | No |
| Hungary | HUN | 0.0003 | No |

MIR indicates mortality-to-incidence ratio.

Table S12. Leave-One-Country-Out analysis: between-country EVT coefficient for ischaemic stroke MIR

| Country | ISO3 | n_countries | n_obs | β_between | SE | p | Δβ |
| --- | --- | --- | --- | --- | --- | --- | --- |
| Lithuania | LTU | 38 | 114 | -0.0154 | 0.0054 | 0.0067 | -0.0027 |
| Greece | GRC | 38 | 114 | -0.0145 | 0.0060 | 0.021 | -0.0017 |
| United Kingdom | GBR | 38 | 114 | -0.0145 | 0.0058 | 0.016 | -0.0017 |
| Sweden | SWE | 38 | 114 | -0.0136 | 0.0056 | 0.020 | -0.0009 |
| Armenia | ARM | 38 | 114 | -0.0136 | 0.0058 | 0.025 | -0.0008 |
| Norway | NOR | 38 | 114 | -0.0135 | 0.0056 | 0.022 | -0.0007 |
| Slovakia | SVK | 38 | 114 | -0.0134 | 0.0062 | 0.038 | -0.0007 |
| Estonia | EST | 38 | 114 | -0.0132 | 0.0057 | 0.027 | -0.0005 |
| Slovenia | SVN | 38 | 114 | -0.0130 | 0.0056 | 0.024 | -0.0003 |
| Italy | ITA | 38 | 114 | -0.0130 | 0.0056 | 0.027 | -0.0002 |
| Ukraine | UKR | 38 | 114 | -0.0129 | 0.0056 | 0.028 | -0.0002 |
| Switzerland | CHE | 38 | 114 | -0.0129 | 0.0056 | 0.025 | -0.0002 |
| Czechia | CZE | 38 | 114 | -0.0129 | 0.0056 | 0.028 | -0.0002 |
| Bosnia & Herz. | BIH | 38 | 114 | -0.0129 | 0.0057 | 0.030 | -0.0001 |
| Moldova | MDA | 38 | 114 | -0.0128 | 0.0055 | 0.025 | -0.0001 |
| Hungary | HUN | 38 | 114 | -0.0128 | 0.0056 | 0.027 | -0.0001 |
| Belgium | BEL | 38 | 114 | -0.0128 | 0.0055 | 0.027 | -0.0000 |
| Netherlands | NLD | 38 | 114 | -0.0128 | 0.0055 | 0.027 | -0.0000 |
| Croatia | HRV | 38 | 114 | -0.0127 | 0.0055 | 0.027 | -0.0000 |
| Latvia | LVA | 38 | 114 | -0.0127 | 0.0056 | 0.028 | -0.0000 |
| Denmark | DNK | 38 | 114 | -0.0127 | 0.0055 | 0.028 | +0.0000 |
| Ireland | IRL | 38 | 114 | -0.0127 | 0.0055 | 0.028 | +0.0001 |
| Finland | FIN | 38 | 114 | -0.0126 | 0.0055 | 0.028 | +0.0001 |
| Malta | MLT | 38 | 114 | -0.0126 | 0.0055 | 0.028 | +0.0001 |
| Poland | POL | 38 | 114 | -0.0126 | 0.0056 | 0.031 | +0.0002 |
| France | FRA | 38 | 114 | -0.0126 | 0.0055 | 0.028 | +0.0002 |
| N. Macedonia | MKD | 38 | 114 | -0.0125 | 0.0056 | 0.031 | +0.0002 |
| Austria | AUT | 38 | 114 | -0.0125 | 0.0055 | 0.029 | +0.0002 |
| Portugal | PRT | 38 | 114 | -0.0125 | 0.0065 | 0.061 | +0.0002 |
| Albania | ALB | 38 | 114 | -0.0124 | 0.0054 | 0.028 | +0.0003 |
| Romania | ROU | 38 | 114 | -0.0124 | 0.0057 | 0.038 | +0.0003 |
| Bulgaria | BGR | 38 | 114 | -0.0123 | 0.0060 | 0.046 | +0.0004 |
| Luxembourg | LUX | 38 | 114 | -0.0123 | 0.0056 | 0.036 | +0.0005 |
| Georgia | GEO | 38 | 114 | -0.0121 | 0.0056 | 0.036 | +0.0006 |
| Israel | ISR | 38 | 114 | -0.0121 | 0.0055 | 0.036 | +0.0006 |
| Germany | DEU | 38 | 114 | -0.0121 | 0.0056 | 0.039 | +0.0007 |
| Montenegro | MNE | 38 | 114 | -0.0118 | 0.0056 | 0.041 | +0.0010 |
| Spain | ESP | 38 | 114 | -0.0111 | 0.0053 | 0.041 | +0.0016 |
| Serbia | SRB | 38 | 114 | -0.0089 | 0.0041 | 0.036 | +0.0038 |

EVT indicates endovascular thrombectomy; MIR, mortality-to-incidence ratio; SE, standard error.

Table S13. Between-country association of IVT rate with stroke burden endpoints (within–between model), reported as a comparator exposure on its own analytic sample. IVT and EVT coefficients were not formally compared

| Endpoint | Between β (95% CI) | Between p | n obs | n countries |
| --- | --- | --- | --- | --- |
| All-stroke MIR | 0.0003 [-0.0030, 0.0037] | 0.84 | 72 | 36 |
| All-stroke YLLs | 5.8359 [-19.7179, 31.3898] | 0.65 | 72 | 36 |
| All-stroke YLDs | 1.1189 [0.2820, 1.9558] | 0.010 | 72 | 36 |
| All-stroke DALYs | 6.9548 [-19.0101, 32.9198] | 0.59 | 72 | 36 |
| Ischaemic stroke MIR | 0.0008 [-0.0029, 0.0045] | 0.67 | 72 | 36 |
| Ischaemic stroke YLLs | 8.2571 [-12.9915, 29.5057] | 0.44 | 72 | 36 |
| Ischaemic stroke YLDs | 1.0915 [0.3073, 1.8758] | 0.0077 | 72 | 36 |
| Ischaemic stroke DALYs | 9.3486 [-12.2762, 30.9735] | 0.39 | 72 | 36 |

CI indicates confidence interval; DALYs, disability-adjusted life-years; EVT, endovascular thrombectomy; IVT, intravenous thrombolysis; MIR, mortality-to-incidence ratio; YLDs, years lived with disability; YLLs, years of life lost.

Table S14. Between-country association of CT scanner density with stroke burden endpoints (within–between model)

| Endpoint | Between β (95% CI) | Between p | n obs | n countries |
| --- | --- | --- | --- | --- |
| All-stroke MIR | 0.0210 [-0.0395, 0.0814] | 0.49 | 117 | 39 |
| All-stroke YLLs | 384.8843 [-178.6207, 948.3893] | 0.17 | 117 | 39 |
| All-stroke YLDs | 19.4889 [-19.6872, 58.6649] | 0.32 | 117 | 39 |
| All-stroke DALYs | 404.3732 [-192.2293, 1000.9758] | 0.18 | 117 | 39 |
| Ischaemic stroke MIR | 0.0172 [-0.0480, 0.0823] | 0.60 | 117 | 39 |
| Ischaemic stroke YLLs | 277.6775 [-195.9468, 751.3018] | 0.24 | 117 | 39 |
| Ischaemic stroke YLDs | 18.1664 [-17.5915, 53.9244] | 0.31 | 117 | 39 |
| Ischaemic stroke DALYs | 295.8439 [-208.4831, 800.1710] | 0.24 | 117 | 39 |

CI indicates confidence interval; CT, computed tomography; DALYs, disability-adjusted life-years; MIR, mortality-to-incidence ratio; YLDs, years lived with disability; YLLs, years of life lost.

Table S15. EVT activity against the two GBD components of ischaemic stroke MIR separately, against MIR conditional on the incidence rate, and against the log death rate conditional on the log incidence rate. Primary three-wave within–between design otherwise (SDI + age 65+, wave fixed effects, country-clustered SE); EVT coefficients fully standardised (SD of the outcome per SD of EVT). Incidence-covariate coefficients are unstandardised; in the log–log model the coefficient is an elasticity

| Outcome | n countries | n obs | EVT between β (95% CI) | Between p | EVT within β (95% CI) | Within p | Incidence covariate β (p) |
| --- | --- | --- | --- | --- | --- | --- | --- |
| Incidence rate per 100 000 | 39 | 117 | -0.24 [-0.67, 0.19] | 0.27 | -0.05 [-0.11, 0.01] | 0.082 | – |
| Death rate per 100 000 | 39 | 117 | -0.41 [-0.80, -0.03] | 0.038 | -0.01 [-0.12, 0.10] | 0.85 | – |
| MIR, adjusted for incidence rate | 39 | 117 | -0.33 [-0.67, 0.02] | 0.061 | 0.12 [-0.04, 0.27] | 0.14 | 0.0010 (0.0029) |
| log death rate, adjusted for log incidence rate | 39 | 117 | -0.16 [-0.32, 0.01] | 0.062 | 0.05 [-0.02, 0.13] | 0.18 | 1.2324 (<0.0001) |

CI indicates confidence interval; EVT, endovascular thrombectomy; GBD, Global Burden of Disease; MIR, mortality-to-incidence ratio; SD, standard deviation; SDI, Socio-Demographic Index; SE, standard error.

Table S16. Model-form sensitivity for the primary EVT–MIR model. A is the specification as fitted (pooled OLS with the EVT rate split into a country mean and a deviation, SDI and age 65+ entering undecomposed, wave fixed effects, finite-sample-corrected country-clustered SE, no random intercept); B also splits SDI and age 65+; C adds a country random intercept to B (correlated random-effects GLS). EVT coefficients fully standardised

| Model | Specification | EVT between β (95% CI) | Between p | EVT within β (95% CI) | Within p | SDI β (p) | Proportion of residual variance attributable to the country intercept (ICC), % |
| --- | --- | --- | --- | --- | --- | --- | --- |
| A | Pooled OLS; EVT decomposed, SDI and age 65+ undecomposed (primary model) | -0.42 [-0.79, -0.05] | 0.027 | 0.10 [-0.07, 0.26] | 0.24 | -1.30 (0.0024) | – |
| B | Pooled OLS; EVT, SDI and age 65+ all decomposed | -0.42 [-0.79, -0.05] | 0.028 | 0.10 [-0.03, 0.23] | 0.12 | within +0.63 (0.86); between -1.30 (0.0026) | – |
| C | Correlated random effects: B with a country random intercept | -0.42 [-0.78, -0.06] | 0.023 | 0.10 [-0.02, 0.23] | 0.11 | within +0.63 (0.86); between -1.30 (0.0016) | 95% |

CI indicates confidence interval; EVT, endovascular thrombectomy; MIR, mortality-to-incidence ratio; OLS, ordinary least squares; SDI, Socio-Demographic Index; SE, standard error.

Table S17. Completeness of the 2023 Stroke Service Tracker admission counts used to convert mechanical thrombectomy percentages to population rates, for the 27 reporting countries. Registry coverage is the value each country reported to the Tracker (the United Kingdom’s is the admission-weighted mean of its four nations). GBD incident ischaemic strokes are the GBD 2023 incidence rate multiplied by population. Differences between reported admissions and GBD incident case counts may reflect registry coverage, recurrent admissions, differences in case definitions and ascertainment, and differences between reported admissions and modelled incidence. The ratio is descriptive rather than a validation measure, and reported coverage does not order it: Bulgaria reports 11% coverage with a ratio of 1.87, Israel 86% with 1.91

| Country | ISO3 | Registry coverage (%) | Ischaemic stroke admissions | GBD incident ischaemic strokes | Admissions / GBD cases | EVT per 100 000 |
| --- | --- | --- | --- | --- | --- | --- |
| Armenia | ARM | 38 | 5,098 | – | – | 14.6 |
| Austria | AUT | 100 | 13,300 | 11,927 | 1.12 | 12.6 |
| Belgium | BEL | not reported | 22,033 | 12,045 | 1.83 | 17.3 |
| Bulgaria | BGR | 11 | 44,513 | 23,828 | 1.87 | 1.0 |
| Czechia | CZE | 100 | 15,785 | 15,056 | 1.05 | 15.2 |
| Denmark | DNK | 81 | 10,675 | 6,549 | 1.63 | 11.9 |
| Estonia | EST | 100 | 2,954 | 1,816 | 1.63 | 16.0 |
| Finland | FIN | 100 | 11,457 | 8,820 | 1.30 | 11.5 |
| Germany | DEU | 100 | 219,894 | 168,549 | 1.30 | 24.8 |
| Hungary | HUN | not reported | 20,000 | 18,288 | 1.09 | 18.6 |
| Israel | ISR | 86 | 11,183 | 5,851 | 1.91 | 8.8 |
| Italy | ITA | 93 | 67,894 | 75,206 | 0.90 | 13.5 |
| Latvia | LVA | 100 | 5,891 | 5,674 | 1.04 | 12.2 |
| Lithuania | LTU | 100 | 8,190 | 6,620 | 1.24 | 28.1 |
| Moldova | MDA | 100 | 3,172 | 4,434 | 0.72 | 6.3 |
| N. Macedonia | MKD | 100 | 2,305 | 6,427 | 0.36 | 0.4 |
| Netherlands | NLD | 92 | 33,736 | 21,498 | 1.57 | 16.3 |
| Norway | NOR | 100 | 7,669 | 5,802 | 1.32 | 8.9 |
| Poland | POL | 100 | 74,662 | 61,343 | 1.22 | 13.3 |
| Portugal | PRT | not reported | 23,273 | 18,576 | 1.25 | 28.6 |
| Romania | ROU | not reported | 40,330 | 49,204 | 0.82 | 2.4 |
| Slovakia | SVK | 100 | 9,028 | 8,694 | 1.04 | 18.8 |
| Spain | ESP | 100 | 68,683 | 56,856 | 1.21 | 13.5 |
| Sweden | SWE | 97 | 17,207 | 17,501 | 0.98 | 13.8 |
| Switzerland | CHE | 56 | 10,706 | 7,922 | 1.35 | 17.1 |
| Ukraine | UKR | 100 | 115,061 | 99,938 | 1.15 | 4.1 |
| United Kingdom | GBR | 100 | 90,011 | 65,676 | 1.37 | 5.3 |

EVT indicates endovascular thrombectomy; GBD, Global Burden of Disease.

## Supplementary Figures

Figure S1. Leave-one-country-out (LOCO) coefficient stability for the between-country EVT association with ischaemic stroke MIR. Each point shows the between-country coefficient when one country is excluded; horizontal bars denote 95% confidence intervals. The dashed line marks the full-sample estimate and the solid grey line marks zero; 38 of the 39 intervals exclude zero, the exception being the model that drops Portugal. Each of the 39 models is the primary within–between fit (Mundlak parameterisation) with wave fixed effects, adjusted for Socio-Demographic Index and proportion aged ≥65 years, with finite-sample-corrected country-clustered standard errors; every model is estimated on 38 countries and 114 country-year observations, against 39 and 117 for the full sample. Drawn in Python with matplotlib from the analysis outputs; no image was acquired or edited outside these tools. EVT=endovascular thrombectomy; MIR=mortality-to-incidence ratio; SE=standard error. *Alt text.* Forest plot with one row per country. Each row shows the between-country coefficient estimated with that country excluded, as a point with a horizontal 95% confidence interval, against a dashed vertical line at the full-sample estimate and a solid line at zero.

Figure S2. Coefficient stability plot: EVT rate coefficient (β) for ischaemic stroke MIR across model specifications. Pooled OLS is shown as the overall association; the within–between model is shown separately for its between- and within-country components. Two-way fixed-effects, first-difference, and fixed-effects-without-2020 estimates are within-country specifications. Points indicate point estimates; horizontal bars denote 95% confidence intervals. The dashed line marks zero. The specifications differ in estimator and therefore in analytic sample: pooled ordinary least squares, the within–between decomposition and two-way fixed effects each use 39 countries and 117 country-year observations; the fixed-effects specification excluding the 2020 wave uses 39 and 78; the three first-difference models use 39 countries and 39 differenced observations each, one per wave pair. All models except the first-difference ones use finite-sample-corrected country-clustered standard errors; the first-difference models use HC1 heteroscedasticity-consistent standard errors, because at most one differenced observation enters per country in each wave pair. Drawn in Python with matplotlib from the analysis outputs; no image was acquired or edited outside these tools. EVT=endovascular thrombectomy; FE=fixed effects; MIR=mortality-to-incidence ratio; SE=standard error. *Alt text.* Forest plot with one row per model specification. Each row shows the EVT-rate coefficient as a point with a horizontal 95% confidence interval, against a vertical reference line at zero.
