# Supplementary material for: Endovascular thrombectomy activity as a candidate marker of organised acute stroke-service capacity: a global burden of disease-based European panel study
Source: Eur Stroke J. 2026 Oct 15;11(10):aakag121. doi: 10.1093/esj/aakag121 (PMC13671229; doi:10.1093/esj/aakag121)
Supplement: Supplementary file 1 [file supplementary_materials_aakag121.zip › GATHER_checklist.docx]

**GATHER Statement — Guidelines for Accurate and Transparent Health Estimates Reporting**

Endovascular thrombectomy activity as a candidate marker of organised acute stroke-service capacity: a Global Burden of Disease-based European panel study

| **Section** | **Item No** | **Recommendation** | **Reported on** |
| --- | --- | --- | --- |
| **Objectives and funding** |  |  |  |
|  | 1 | Define the indicator(s), populations, and time periods for which estimates were made. | Methods, Study design; Methods, Data sources. Indicators: country-level EVT rate (procedures per 100 000 population/year) as the exposure; ischaemic stroke mortality-to-incidence ratio (MIR; primary outcome), ischaemic stroke YLLs, DALYs, and YLDs per 100 000 (secondary) as outcomes, all from the GBD 2023 release. Populations: 39 European and adjacent countries (national level, both sexes, all ages); 27 of these 39 countries additionally contributed wave-4 (2023) data. Time periods: primary three-wave panel 2016, 2019, 2020 (117 country-year observations); secondary four-wave extension adds 2023 (144 country-year observations). |
|  | 2 | List the funding sources for the work. | Declaration in main text: "There was no funding source for this study." Authors had full data access and final responsibility for submission. |
| **Data inputs (sources of empirical data)** |  |  |  |
|  | 3 | Describe how data were identified and how data were accessed. | Methods, Data sources. EVT rates: Aguiar de Sousa surveys (waves 1-3, open-access publications) and the European Stroke Organisation Stroke Action Plan for Europe Stroke Service Tracker (wave 4). The Stroke Service Tracker country-year aggregates are not publicly available; their use in this study was authorised by the ESO Stroke Action Plan committee, and they can be requested, with permission, from the European Stroke Organisation. GBD estimates: GBD 2023 release, accessed via the GBD Results Tool. Other covariates: IAEA IMAGINE database, OECD Health Statistics, WHO Global Health Observatory, WHO Global Health Expenditure Database, World Bank Development Indicators (all openly available from their respective publishers). |
|  | 4 | Specify the inclusion and exclusion criteria. Identify all ad-hoc exclusions. | Methods, Data sources. Inclusion: European and adjacent countries with EVT rate available in any survey wave. Wave-4 (2023) inclusion required the Stroke Service Tracker to carry both a reported MT percentage and a non-zero ischaemic admission count; the rate is undefined without either. A reported MT percentage of zero would have been treated as missing rather than as a true zero where the same country had recorded an Aguiar wave-3 EVT rate of ≥1 per 100 000, and retained as a genuine zero otherwise; no country reported a zero MT percentage in 2023, so this prespecified rule changed no country's classification. Twelve of the 39 countries were excluded from wave 4 on missing data alone. UK constituent nations were aggregated to a single GBR value using admission-weighted percentages. |
|  | 5 | Provide information on all included data sources and their main characteristics (population represented, year(s), measurement method, sample size). | Methods, Data sources (prose summary of each input — population represented, year coverage, measurement method, and source publication); Supplementary Methods, Additional data-source details (per-source notes on coverage and measurement caveats); column-level documentation in data_dictionary.md within the submission package. |
|  | 6 | Identify and describe any categories of input data that have potentially important biases. | Methods, Sensitivity and extension analyses (waves 1-3 from Aguiar surveys vs. wave 4 from a different administrative reporting instrument); Discussion, Limitations (reporting selection, GBD model uncertainty not propagated, source-change conflated with 2023 period effect). |
|  | 7 | Describe and give sources for any other data inputs (non-empirical: expert judgement, derived quantities). | Methods, Data sources. Adjustment variables: Socio-Demographic Index (GBD 2023 release composite) and proportion of population aged ≥65 years (World Bank Development Indicators). Derived quantities used in this study: (i) ischaemic stroke MIR computed as the GBD 2023 deaths-rate to incidence-rate ratio; (ii) EVT rate for the 2023 wave derived from the ESO Stroke Service Tracker as MT% / 100 × ischaemic-stroke admissions / population × 100 000 (IVT 2023 analogously); (iii) United Kingdom EVT and IVT rates aggregated from constituent-nation values using admission-weighted percentages. |
|  | 8 | Provide all data inputs in a file format from which data can be efficiently extracted, with metadata, or name the institution that retains the right to the data. | Mendeley Data (DOI: 10.17632/r9p47jkz8w.1) holds the analysis code, the machine-readable regression outputs (statistical_results.json) and column-level documentation of every derived country-year variable (data_dictionary.md) under an MIT licence. No derived analytic dataset is deposited, because one input cannot be redistributed: the European Stroke Organisation retains the rights to the Stroke Service Tracker country-year aggregates that supply the 2023 wave, and these are available from the ESO on permission. The remaining inputs are obtainable from their publishers as named in item 3, so the panel can be reassembled from its original sources. See Methods, Data availability. |
| **Data analysis** |  |  |  |
|  | 9 | Provide a conceptual overview of the data analysis method. | Methods, Statistical analysis. Mundlak within-between linear panel model with country-clustered standard errors; comparator framework for parallel acute-care and macro-system exposures; adjusted Mundlak specifications for macro-system overlap. |
|  | 10 | Provide a detailed description of all steps of the analysis, including mathematical formulae. | Methods, Statistical analysis. Mundlak model equation in main text; data cleaning, EVT conversion, UK aggregation, and CT interpolation described in Methods, Data sources. Adjusted EVT models B-E and the source-change sensitivity analyses are specified there as well. Two of the three prespecified source-change analyses were carried out: the source-homogeneous Aguiar-only three-wave panel (which the first protocol amendment made the primary specification) and the balanced 27-country restriction. The third, a binary Aguiar-vs-Stroke-Service-Tracker source indicator, was not: under the primary specification's wave fixed effects it is collinear with the 2023 wave dummy and therefore not separately identifiable. Both amendments predate any inspection of outcome regression results and are recorded in Methods, Changes from the protocol, and in Supplementary Methods, Protocol amendments. |
|  | 11 | Describe how candidate models were evaluated and how the final model(s) were selected. | Methods, Statistical analysis (Mundlak with SDI + age 65+ as the primary model). The original protocol prespecified the unbalanced four-wave Mundlak as primary and the balanced three-wave Aguiar panel as a source-change sensitivity; before inspection of outcome regression results, these two were exchanged so that the source-homogeneous three-wave Aguiar panel became the primary (see Methods, Changes from the protocol; Supplementary Methods, Protocol amendments). Supporting specifications (pooled OLS, two-way FE, first-differences) are reported in the supplement as cross-checks, not as alternative primary models. |
|  | 12 | Provide the results of model performance evaluation and any sensitivity analysis. | Supplementary tables and figures: pooled OLS, two-way FE, first-differences, cross-section, Mundlak sensitivities S1-S5, Cook's distance, leave-one-country-out, comparator-specific Mundlak (CT, IVT). Main-text Results: source-change sensitivities (Aguiar-only, balanced 27) and alternative endpoint (deaths rate). |
|  | 13 | Describe methods for calculating uncertainty of estimates. State which sources of uncertainty were, and were not, accounted for. | Methods, Statistical analysis. Country-clustered robust SE (Liang–Zeger sandwich) with finite-sample corrections for all coefficients. Limitations: GBD model uncertainty intervals are not propagated into our regressions; EVT and ESO administrative reports are subject to unmodelled measurement error. |
|  | 14 | State how analyses take into account non-independence of data. | Methods, Statistical analysis. Country-clustered standard errors (39 clusters) for all regression models, accounting for repeated observations within country. Within-between decomposition formally separates cross-country from within-country variation. |
|  | 15 | Describe any limitations of these methods. State what factors may affect the unconditional generalizability of estimates. | Discussion, Limitations (6 items). (1) Ecological design vulnerable to residual confounding; MIR built from two modelled GBD rates without GBD uncertainty propagated. (2) National EVT activity is an imperfect proxy for service capacity, with interpretation of within-country change further complicated by post-DAWN/DEFUSE-3 late-window thrombectomy eligibility. (3) The 2023 extension is limited by a measurement-source transition and selective wave-4 reporting (motivating the balanced 3-wave primary). (4) Comparator measures are heterogeneous and incompletely longitudinal; correlated macro-system indicators cannot fully separate stroke-service organisation from broader national resources. (5) The observation window includes 2020 and COVID-19 disruption (analyses excluding 2020 yielded a compatible pattern). (6) Sample comprised predominantly European and relatively high-income countries, limiting generalisability of EVT activity as a monitoring marker to lower-reperfusion settings. |
| **Results and discussion** |  |  |  |
|  | 16 | Provide published estimates in a file format from which data can be efficiently extracted. | Mendeley Data (DOI: 10.17632/r9p47jkz8w.1): statistical_results.json, the machine-readable regression outputs behind every reported estimate. The supplementary tables accompany the manuscript as supplementary files; the scripts that generate them, and which also write .csv and .xlsx copies, are in the same deposit. |
|  | 17 | Report a quantitative measure of the uncertainty of estimates. | All reported coefficients carry 95% confidence intervals and p-values (analytic cluster-robust with finite-sample corrections) in main text and tables. |
|  | 18 | Interpret results in light of existing evidence. If updating a previous set of estimates, describe the reasons for changes. | Discussion, paragraphs 2-4; Research-in-context panel. Findings are discussed against the established macro-system gradient (THE, UHC) and against acute-care components (IVT, stroke unit, CT) within a common standardised framework. The study is not an update of a previous estimate set. |
