# Supplementary material for: Endovascular thrombectomy activity as a candidate marker of organised acute stroke-service capacity: a global burden of disease-based European panel study
Source: Eur Stroke J. 2026 Oct 15;11(10):aakag121. doi: 10.1093/esj/aakag121 (PMC13671229; doi:10.1093/esj/aakag121)
Supplement: Supplementary file 1 [file supplementary_materials_aakag121.zip › STROBE_checklist.docx]

**STROBE Statement — Checklist of items for cross-sectional studies**

Endovascular thrombectomy activity as a candidate marker of organised acute stroke-service capacity: a Global Burden of Disease-based European panel study

| **Section** | **Item No** | **Recommendation** | **Reported on** |
| --- | --- | --- | --- |
| Title and abstract | 1 | (a) Indicate study design in the title or the abstract; (b) Provide an informative and balanced summary | Abstract, Methods section (study design described as a repeated cross-sectional panel analysis) |
| **Introduction** |  |  |  |
| Background / rationale | 2 | Explain the scientific background and rationale for the investigation | Introduction, paragraphs 1–3 |
| Objectives | 3 | State specific objectives, including any prespecified hypotheses | Introduction, final paragraph; Study protocol Section 2 |
| **Methods** |  |  |  |
| Study design | 4 | Present key elements of study design early in the paper | Methods, Study design; Methods, Changes from the protocol (primary specification exchanged with a prespecified source-change sensitivity before inspection of outcome regression results — see also Supplementary Methods, Changes Protocol amendments). |
| Setting | 5 | Describe the setting, locations and relevant dates | Methods, Study design + Data sources. Primary panel: 39 European and adjacent countries, 2016/2019/2020 (balanced three-wave Aguiar panel); four-wave extension adds 2023 ESO Stroke Service Tracker wave (27 of 39 countries reporting). |
| Participants | 6 | (a) Give the eligibility criteria, and the sources and methods of selection of participants | Methods, Data sources (panel construction). Primary three-wave panel: 39 countries × 3 waves = 117 country-year observations; four-wave extension: 144 country-year observations with 27 of 39 countries reporting in 2023. |
| Variables | 7 | Clearly define all outcomes, exposures, predictors, potential confounders and effect modifiers; give diagnostic criteria if applicable | Methods, Data sources + Statistical analysis (primary, comparator, adjustment) |
| Data sources / measurement | 8 | For each variable of interest, give sources of data and details of methods of assessment (measurement). Describe comparability of assessment methods if there is more than one group | Methods, Data sources (Aguiar surveys waves 1–3; European Stroke Organisation Stroke Service Tracker wave 4; GBD 2023 release for burden; harmonised country-year master table for comparator covariates). Source change between wave 3 and wave 4 acknowledged in Methods and Discussion. |
| Bias | 9 | Describe any efforts to address potential sources of bias | Methods, Statistical analysis: comparator framework; adjusted EVT models B–E; influence diagnostics (Cook's distance, leave-one-country-out); source-change and reporter-selection sensitivities for the four-wave extension. |
| Study size | 10 | Explain how the study size was arrived at | Methods, Data sources; Study protocol Section 9. Sample defined by countries with valid EVT data across the panel waves; no formal sample-size calculation. |
| Quantitative variables | 11 | Explain how quantitative variables were handled in the analyses. If applicable, describe which groupings were chosen and why | Methods, Statistical analysis. All continuous exposures and outcomes analysed on their natural scale or standardised per 1 SD; no categorisation. |
| Statistical methods | 12a | Describe all statistical methods, including those used to control for confounding | Methods, Statistical analysis (primary Mundlak within-between model on the balanced three-wave Aguiar panel with wave fixed effects and country-clustered robust SE with finite-sample corrections; comparator framework; adjusted models B–E; four-wave extension and its source-change and reporter-selection sensitivities; alternative-endpoint analysis on ischaemic stroke deaths rate). Methods, Changes from the protocol documents the amendment that exchanged the originally prespecified four-wave primary with the three-wave Aguiar sensitivity, made before inspection of outcome regression results. |
|  | 12b | Describe any methods used to examine subgroups and interactions | Not applicable (no formal subgroup or interaction analyses). |
|  | 12c | Explain how missing data were addressed | Methods, Data sources. EVT 2023: a reported MT percentage of zero would have been treated as missing rather than as a true zero where the same country had a non-zero Aguiar wave-3 EVT rate; no country reported a zero MT percentage in 2023, so this prespecified rule reclassified no observations, and all 12 wave-4 exclusions rest on missing data. IVT 2023: equivalent quality filter; IVT 2019 and 2020 use Aguiar wave-3 values. CT scanner density: linear interpolation across panel years where source data covered only some panel years. Stroke unit density: country-level snapshot indicator without wave-specific variation; entered as a between-country comparator only. Listwise deletion in all regression models on the per-analysis covariate set. |
|  | 12d | If applicable, describe analytical methods taking account of sampling strategy | Methods, Statistical analysis. Country-clustered robust standard errors with finite-sample corrections; Mundlak within–between decomposition formally separates between- and within-country variation. |
|  | 12e | Describe any sensitivity analyses | Methods, Statistical analysis (final paragraphs); supplementary tables. Includes pooled OLS, two-way fixed effects, first-difference models, fixed effects excluding 2020, sensitivity covariate sets (S1–S5), Cook's distance, and leave-one-country-out diagnostics. |
| **Results** |  |  |  |
| Participants | 13a | Report the numbers of individuals at each stage of the study | Results, paragraph 1 (39 countries; 117 country-year observations in the primary three-wave panel; 144 country-year observations in the four-wave extension). |
|  | 13b | Give reasons for non-participation at each stage | Results, paragraph 1 + Methods, Data sources: 12 of 39 countries had no valid wave-4 (2023) EVT value, reflecting incomplete ESO Stroke Service Tracker reporting or quality criteria not met. |
|  | 13c | Consider use of a flow diagram | Not applicable (ecological country-level study). |
| Descriptive data | 14a | Give characteristics of study participants (e.g. demographic, clinical, social) and information on exposures and potential confounders | Table 1; Figure 1. |
|  | 14b | Indicate the number of participants with missing data for each variable of interest | Table 1 (n per wave and overall); supplementary tables on per-variable per-wave missingness and CT interpolation audit; interpolation and exclusion rules in Supplementary Methods. |
| Outcome data | 15 | Report numbers of outcome events or summary measures | Table 1 (per-wave summary measures). |
| Main results | 16a | Give unadjusted estimates and, if applicable, confounder-adjusted estimates and their precision (e.g. 95% confidence interval). Make clear which confounders were adjusted for and why they were included | Table 2 (primary Mundlak adjusted for SDI + age 65+); Table 3 (adjusted EVT models on the fully-standardised β scale matching the Results-text and Figure 3); Figure 2 (burden decomposition); Figure 3 (comparator); supplementary tables (pooled OLS, covariate-adjusted with cluster-robust SE). |
|  | 16b | Report category boundaries when continuous variables were categorised | Not applicable (all quantitative variables analysed continuously). |
|  | 16c | If relevant, consider translating estimates of relative risk into absolute risk for a meaningful time period | Not applicable (ecological association, no relative risk). |
| Other analyses | 17 | Report other analyses done (e.g. analyses of subgroups and interactions, sensitivity analyses) | Results, "Robustness of the EVT–MIR association to adjustment" subsection; Results, "All-stroke endpoints and supporting specifications" subsection; supplementary tables and figures. |
| **Discussion** |  |  |  |
| Key results | 18 | Summarise key results with reference to study objectives | Discussion, paragraph 1. |
| Limitations | 19 | Discuss limitations of the study, taking into account sources of potential bias or imprecision. Discuss both direction and magnitude of any potential bias | Discussion, limitations paragraph — six numbered limitations: (1) ecological design vulnerable to residual confounding, with MIR built from two modelled GBD rates and uncertainty not propagated; (2) national EVT activity is an imperfect proxy for service capacity, with within-country interpretation further complicated by post-DAWN/DEFUSE-3 late-window thrombectomy eligibility; (3) the 2023 extension is limited by a measurement-source transition and selective wave-4 reporting; (4) comparator measures are heterogeneous and incompletely longitudinal, and correlated macro-system indicators cannot fully separate stroke-service organisation from broader national resources; (5) the window includes 2020 and COVID-19 disruption (analyses excluding 2020 yielded a compatible pattern); (6) sample comprised predominantly European and relatively high-income countries, limiting generalisability. |
| Interpretation | 20 | Give a cautious overall interpretation considering objectives, limitations, multiplicity of analyses, results from similar studies and other relevant evidence | Discussion, interpretation paragraphs. EVT activity interpreted as a candidate country-level marker of organised acute stroke-service capacity; the ecological analysis does not estimate the causal effect of expanding EVT provision on population stroke outcomes. |
| Generalisability | 21 | Discuss the generalisability (external validity) of the study results | Discussion, limitations (predominantly European, high-income setting). |
| **Other information** |  |  |  |
| Funding | 22 | Give the source of funding and the role of the funders for the present study and, if applicable, for the original study on which the present article is based | Methods, Role of the funding source ("There was no funding source for this study"). |
