# Supplementary material for: Endovascular thrombectomy activity as a candidate marker of organised acute stroke-service capacity: a global burden of disease-based European panel study
Source: Eur Stroke J. 2026 Oct 15;11(10):aakag121. doi: 10.1093/esj/aakag121 (PMC13671229; doi:10.1093/esj/aakag121)
Supplement: Supplementary file 1 [file supplementary_materials_aakag121.zip › study_protocol.docx]

# Study Protocol

**Endovascular thrombectomy activity as a candidate marker of organised acute stroke-service capacity: a GBD-based European panel study**

***The protocol is reproduced as prespecified. The subsequently documented amendments to the analysis hierarchy are described in the main manuscript and Supplementary Methods, and were made before inspection of outcome regression results.***

## 1. Background and rationale

National endovascular thrombectomy (EVT) activity varies widely across European and adjacent countries. EVT requires the coordinated functioning of an entire chain of acute stroke care: prehospital triage, imaging-based large-vessel-occlusion detection, transfer pathways, angiosuite access, neurointerventional workforce, stroke-unit and intensive care, and post-acute coordination. Country-level EVT activity may therefore behave as a measurable cross-national indicator of mature acute stroke-system capacity. Whether the EVT–outcome cross-national association is distinguishable from associations seen with individual upstream acute stroke-care components (intravenous thrombolysis rate, CT scanner density, stroke unit density) or with broader macro-system indicators (national health expenditure, Universal Health Coverage service coverage, income inequality), and whether it is endpoint-specific to mortality-related ischaemic stroke burden rather than to disability-only outcomes, requires a panel design with formal between- versus within-country decomposition and a common comparator framework.

## 2. Objectives

**Primary objective.** To determine whether national EVT activity is associated with ischaemic stroke mortality-to-incidence ratio (MIR) across countries, separating between-country from within-country associations using a Mundlak within–between decomposition.

**Secondary objectives.**

1. To examine whether the between-country EVT association is observed for the mortality-related ischaemic stroke burden endpoints (years of life lost [YLLs], disability-adjusted life-years [DALYs]) and to test the endpoint specificity by contrasting with the disability-only ischaemic stroke years lived with disability (YLDs).
2. To compare the EVT signal with that of parallel acute stroke-care indicators (IVT rate, CT scanner density, stroke unit density) and broader macro-system indicators (total health expenditure per capita, Universal Health Coverage service coverage index, Gini index of income inequality) within a common Mundlak framework standardised per 1 standard deviation higher exposure.
3. To test whether the between-country EVT–MIR association persists after adjustment for the macro-system indicators (THE per capita, UHC service coverage, jointly and separately) and after adjustment for the parallel acute stroke-care indicators.
4. To examine whether the cross-national EVT–MIR association is sensitive to the change of EVT exposure source between waves 3 (Aguiar de Sousa surveys for 2016/2019/2020) and 4 (ESO Stroke Service Tracker for 2023) and to the selection of countries reporting 2023 data.
5. To examine the all-stroke endpoints (MIR, YLLs, DALYs, YLDs) and the GBD ischaemic stroke deaths rate per 100,000 as contextual or alternative endpoints in the appendix.

## 3. Study design

Repeated cross-sectional ecological analysis at the country level over four survey waves (2016, 2019, 2020, 2023).

## 4. Study population

Thirty-nine European and adjacent countries contributed to the primary four-wave panel (2016, 2019, 2020, 2023; 144 country-year observations), with 27 of the 39 countries reporting valid 2023 EVT data and the remaining 12 countries contributing waves 1–3 only. The balanced three-wave Aguiar panel (2016, 2019, 2020; 117 country-year observations) is a prespecified source-change sensitivity analysis (see §8). Inclusion in wave 4 required the country to report a mechanical thrombectomy percentage and a non-zero ischaemic stroke admission count in the European Stroke Organisation Stroke Service Tracker; reports of zero mechanical thrombectomy in countries that recorded an EVT rate of at least 1 per 100,000 in Aguiar wave 3 were treated as missing rather than as true zeros, while zeros in countries without prior recorded activity were retained.

## 5. Data sources

**Exposure.** National EVT rates per 100,000 population per year. Waves 1–3 (reference years approximately 2016, 2019, 2020) from Aguiar de Sousa et al., *European Stroke Journal* 2019 and 2023. Wave 4 (reference year 2023) from the European Stroke Organisation Stroke Action Plan for Europe Stroke Service Tracker; mechanical thrombectomy percentages converted to procedures per 100,000 population using country- and year-matched ischaemic stroke admission counts and total population values from the same source. The exposure-source change between wave 3 and wave 4 is acknowledged in the analysis plan and addressed in targeted source-change sensitivity analyses.

**Outcomes.** Year-matched ischaemic stroke and all-stroke burden estimates from the Global Burden of Disease (GBD) Study 2023 release, accessed via the GBD Results Tool. For both ischaemic stroke and all stroke we extract deaths, incidence, YLLs, YLDs, and DALYs per 100,000 population (Rate metric, both sexes, all ages). MIR is defined as the ratio of the deaths rate to the incidence rate. All four panel waves use this single GBD release to avoid contamination of within-country comparisons by release-to-release methodology revisions.

**Comparator and adjustment covariates.** Intravenous thrombolysis (IVT) rate per 100,000, stroke unit density per million, total health expenditure (THE) per capita in current USD, Universal Health Coverage (UHC) service coverage index, and the Gini index of income inequality — drawn from a harmonised country-year master table (sources: European Stroke Organisation surveys, WHO Global Health Expenditure Database, WHO/World Bank UHC tracking, World Bank Development Indicators). For the primary four-wave comparator analysis, IVT rate is available for 2019, 2020 (Aguiar surveys) and 2023 (European Stroke Organisation Stroke Service Tracker, computed analogously to the EVT 2023 derivation as IVT% / 100 × ischaemic admissions / population × 100,000). IVT therefore enters the primary comparator with limited within-country variation, and inherits the same Aguiar-to-Tracker source change as EVT 2023; analyses restricted to the balanced three-wave Aguiar panel use the 2019 and 2020 Aguiar values only (between-country exposure without within-country variation). Stroke unit density is available only as a country-level snapshot indicator without wave-specific variation; we therefore analyse it only as a between-country comparator. CT scanner density (scanners per 100,000 population) was compiled from IAEA IMAGINE, OECD Health Statistics, and WHO Global Health Observatory, with linear interpolation for country-years with missing data.

**Primary adjustment covariates.** Socio-Demographic Index (SDI; GBD 2023 release) and proportion of population aged 65 years or older (World Bank Development Indicators).

## 6. Primary endpoint

Ischaemic stroke mortality-to-incidence ratio (MIR), defined as the ratio of the deaths rate to the incidence rate within each country-year, from the GBD 2023 release.

## 7. Secondary endpoints

Reported in the main text alongside the primary endpoint:

1. Ischaemic stroke YLLs per 100,000.
2. Ischaemic stroke DALYs per 100,000.
3. Ischaemic stroke YLDs per 100,000 (disability-only contrast endpoint to test the specificity of the EVT signal to mortality-related burden).

Reported in the appendix as contextual or alternative outcomes:

4. All-stroke MIR, YLLs, DALYs, YLDs.
5. Ischaemic stroke deaths rate per 100,000 (alternative to MIR; avoids the MIR ratio of two modelled GBD rates).

## 8. Statistical analysis plan

**Primary model.** Mundlak within–between decomposition with country-clustered standard errors. The EVT rate is decomposed into a between-country component (country-level time-averaged mean) and a within-country component (deviation from that mean); adjustment covariates are SDI and proportion aged ≥65.

Inference. Country-level clustering (39 clusters) with finite-sample corrections (use_correction=True, df_correction=True) and t-distribution inference. The primary specification includes wave (year) fixed effects to absorb common time trends, so the within-country EVT coefficient reflects deviations from the panel-wide temporal mean rather than the joint upward trend in EVT and downward trend in MIR across waves. Single inferential measure: analytic cluster-robust p-value with the above corrections; no supplementary inference procedure is reported.

**Standardisation.** For the primary endpoint forest figure, between- and within-country EVT coefficients are presented fully standardised (per 1 SD higher EVT, expressed in SD of the endpoint), so that magnitudes are directly comparable across endpoints with different natural units. For the comparator-exposure figure, exposures are standardised per 1 SD of the exposure, and the outcome (ischaemic MIR) is also standardised to its own SD so the x-axis is in standardised effect-size units.

**Comparator exposure framework.** Each of the six additional exposures (IVT rate, CT scanner density, stroke unit density, THE per capita, UHC service coverage, Gini index) is fitted in the same Mundlak framework against ischaemic stroke MIR, with the same SDI + age65 adjustment (except where the comparator is itself one of these adjustment variables) and standardised per 1 SD of the exposure (computed in its own analytic sample). Stroke unit density enters the primary four-wave comparator as a between-country exposure only (country-level snapshot without wave-specific variation). IVT rate is available in the primary four-wave panel for 2019, 2020 (Aguiar surveys) and 2023 (Stroke Service Tracker, derived analogously to the EVT 2023 figure); it inherits the same Aguiar-to-Tracker source change as EVT 2023. The balanced three-wave Aguiar sensitivity restricts IVT to the 2019 and 2020 Aguiar values (between-country exposure only).

**Adjusted EVT models.** Four adjusted Mundlak specifications in addition to the primary model test whether the EVT–MIR association is distinguishable from broader health-system resource gradients: (B) replacing SDI with THE per capita; (C) replacing SDI with UHC service coverage; (D) adjusting for THE and UHC jointly (reported as a stringent rather than primary specification owing to collinearity between THE, UHC, and SDI); and (E) adjusting for IVT rate, stroke unit density, and CT scanner density alongside SDI (acute-process adjustment).

**Source-change sensitivity analyses.** Four targeted sensitivity analyses address source-change and 2023-reporting selection: (i) Mundlak restricted to the Aguiar-only three-wave panel (2016/2019/2020); (ii) Mundlak restricted to the 27 countries with valid 2023 EVT data across all four waves; (iii) four-wave Mundlak with a binary indicator distinguishing Aguiar (2016/2019/2020) from Stroke Service Tracker (2023) observations; and (iv) descriptive comparison of the 27 wave-4 reporters versus the 12 non-reporters on pre-2023 EVT activity, ischaemic MIR, SDI, and age structure.

**Alternative-endpoint sensitivity.** The primary Mundlak model is additionally fitted with the GBD ischaemic stroke deaths rate per 100,000 as the dependent variable (replacing MIR), to test whether the primary finding is sensitive to the MIR being a ratio of two modelled GBD rates.

**Supporting specifications.** Pooled OLS with country-clustered standard errors; two-way (country and time) fixed effects with entity-clustered standard errors; first-difference models on the Aguiar-internal wave pairs (2016–2019, 2019–2020, 2016–2020), excluding the cross-source 2020–2023 pair; two-way fixed-effects model excluding the 2020 wave (COVID-19 sensitivity).

**Confounder-set sensitivity analyses.** Addition of the Gini index; replacement of SDI with UHC or THE; inclusion of IVT rate and stroke unit density as additional covariates; population-weighted pooled OLS.

**Influence diagnostics.** Cook’s distance and leave-one-country-out (LOCO) analysis, reported in the appendix.

**Software.** Python 3.13 with statsmodels 0.14.5, linearmodels 7.0 (for panel fixed-effects estimation), scipy 1.16.2, pandas 2.3.2, numpy 2.3.3, matplotlib 3.10.6 (figures), and geopandas 1.1.3 (country map). The submission folder includes a requirements.txt pinning these versions; the submitted figure PDFs embed the matplotlib 3.10.6 metadata signature so the rendering toolchain can be verified directly from the figure files.

## 9. Sample size

No formal sample size calculation. The primary four-wave panel comprises the 39 European and adjacent countries observed across 2016, 2019, 2020 and 2023 (144 country-year observations; 27 of 39 countries reporting valid 2023 EVT data). The balanced three-wave Aguiar sensitivity comprises the same 39 countries observed in 2016, 2019 and 2020 only (117 country-year observations). Comparator analyses operate on smaller samples reflecting comparator availability (see Methods).

## 10. Multiplicity

One primary endpoint (ischaemic stroke MIR) and three ischaemic secondary endpoints (YLLs, DALYs, YLDs) reported in the main text; four all-stroke endpoints reported in the appendix. The comparator framework adds six between-country comparator exposures evaluated against the primary endpoint; the adjusted-models block adds four adjusted specifications of the EVT-on-MIR estimate. No formal multiplicity adjustment is applied. Secondary endpoints, comparator analyses, and adjusted specifications are interpreted as supporting evidence for the consistency, specificity, and robustness of the primary finding rather than as independent confirmatory tests.

## 11. Ethics

This study uses aggregated, publicly available country-level data. No individual-level data were collected or analysed. Ethics approval was not required.

## 12. Reporting

The study is reported in accordance with the STROBE guidelines for observational studies (cross-sectional checklist).
